# Supplementary material for: The Trypanosoma cruzi Antigen and Epitope Atlas: antibody specificities in Chagas disease patients across the Americas
Source: Nat Commun. 2023 Apr 3;14:1850. doi: 10.1038/s41467-023-37522-9 (PMC10070320; doi:10.1038/s41467-023-37522-9)
Supplement: Supplementary file 1 — Supplementary Information [file 41467_2023_37522_MOESM1_ESM.pdf]

SUPPLEMENTARY INFORMATION FOR

The Chagas Antigen and Epitope Atlas

Alejandro D. Ricci, Leonel Bracco, Emir Salas-Sarduy, Janine M. Ramsey, Melissa S. Nolan, M. Katie Lynn, Jaime Altcheh, Griselda E Ballering, Faustino Torrico, Norival Kesper, Juan C. Villar, Iván S. Marcipar, Jorge D. Marco, Fernán Agüero*

*** Corresponding author. Email:** fernan@iib.unsam.edu.ar

This PDF file includes:

- Supplementary Figures S1 to S15

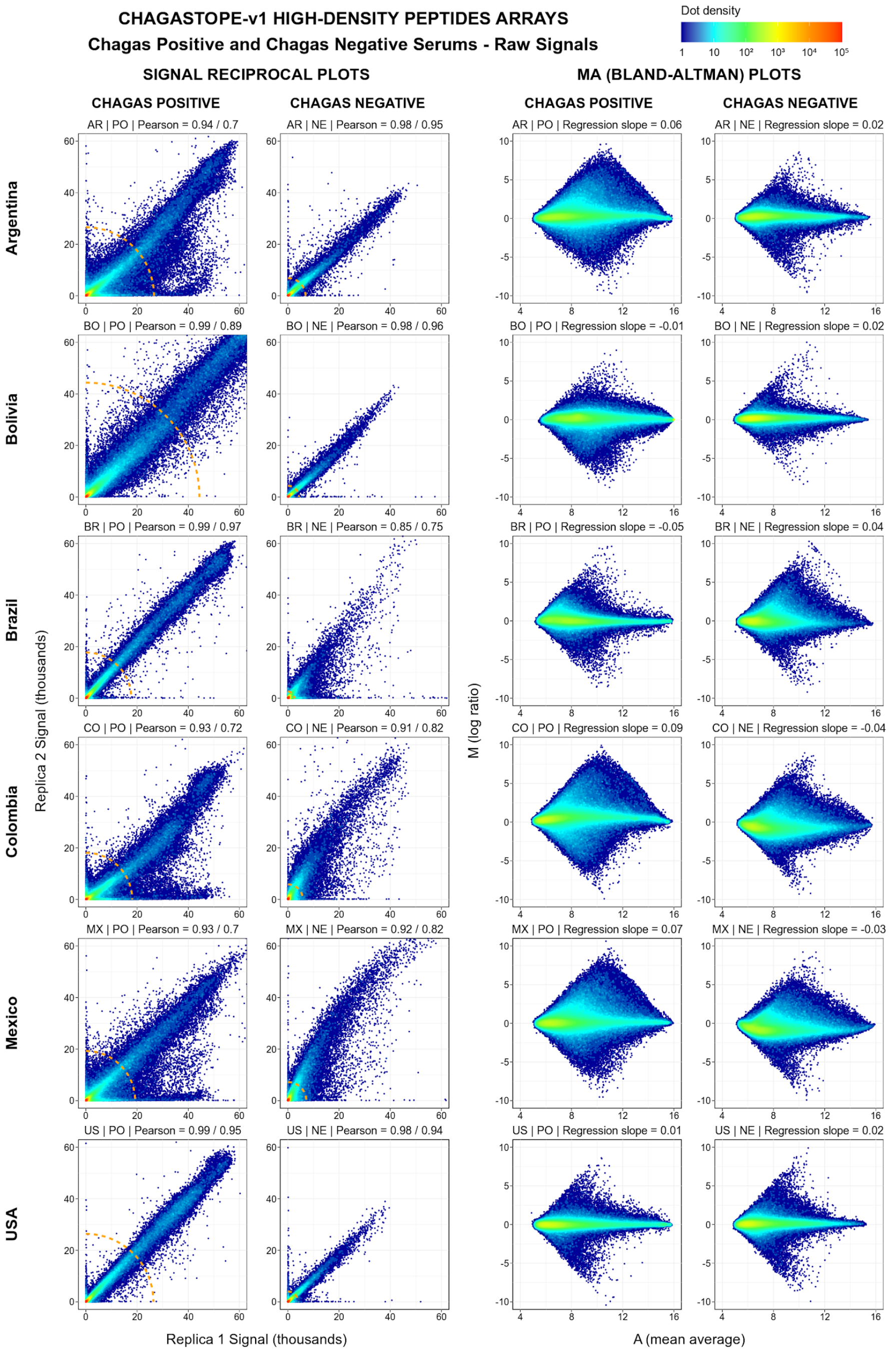

Supplementary Figure S1a. Quality control of 1-plex microarray assays. *Each biological sample (serum pool) was assayed in duplicate (technical replicates). The signal correlation between technical replicates is shown both in reciprocal plots and in MA (Bland-Altman) plots. Each point represents one unique peptide or addressable array spot (n = 2,842,420). The density is shown in a color gradient (see key in figure). In the reciprocal plots, the raw signal data of one replicate is plotted against the second replicate and two Pearson scores are calculated: the first one using all peptides and the second one using only the ones with the top 1% signals, which are those outside the dashed orange line. In MA plots, each signal is replaced by its log2 and then the average signal of the two replicates (A) is plotted against the difference of the two signals (M).*

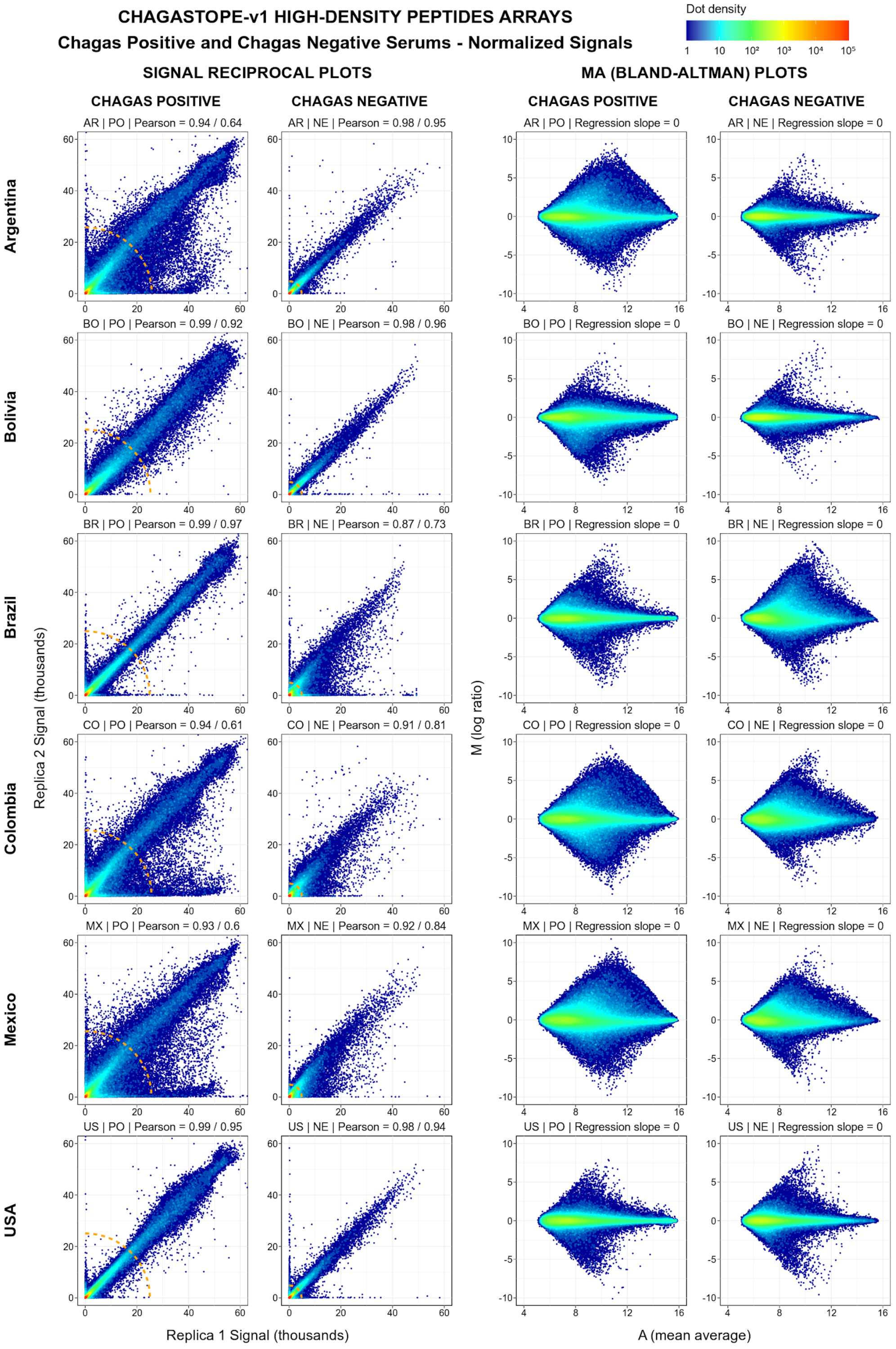

Supplementary Figure S1b. Quality control of 1-plex microarray assays after normalization. *The legend is similar to that in Supplementary Figure S1a, but for the normalised signals.*

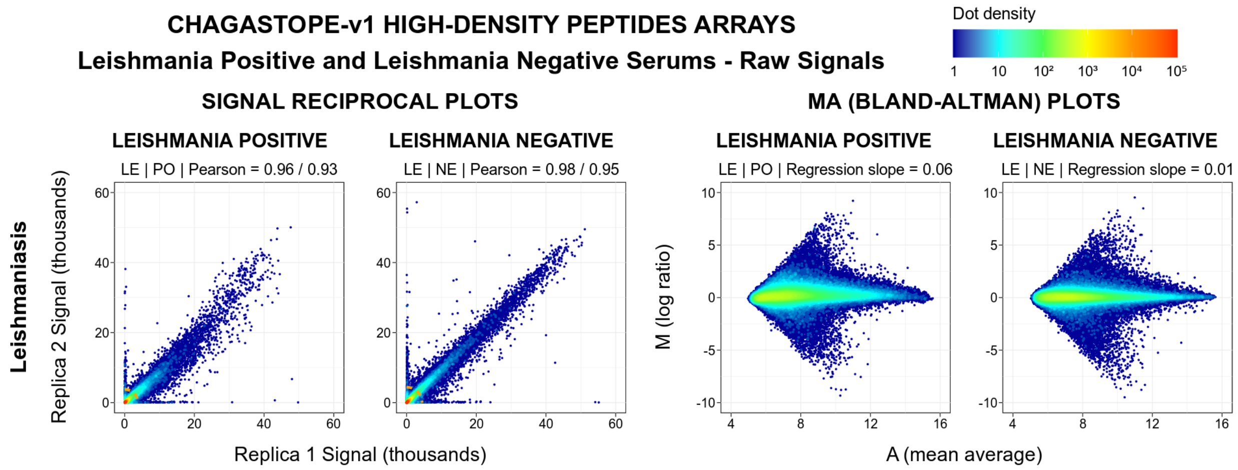

Supplementary Figure S1c. Quality control of 1-plex microarray assays for Leishmania. *The legend is similar to that in Supplementary Figure S1a, but for the Leishmania positive and negative controls.*

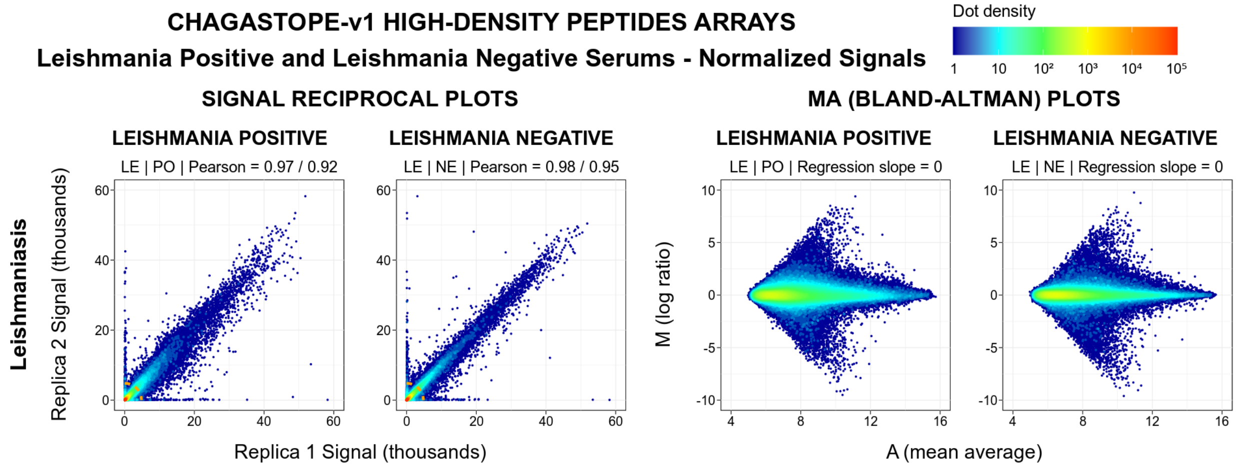

Supplementary Figure S1d. Quality control of 1-plex microarray assays for Leishmania after normalization. *The legend is similar to that in Supplementary Figure S1c, but for the normalised signals.*

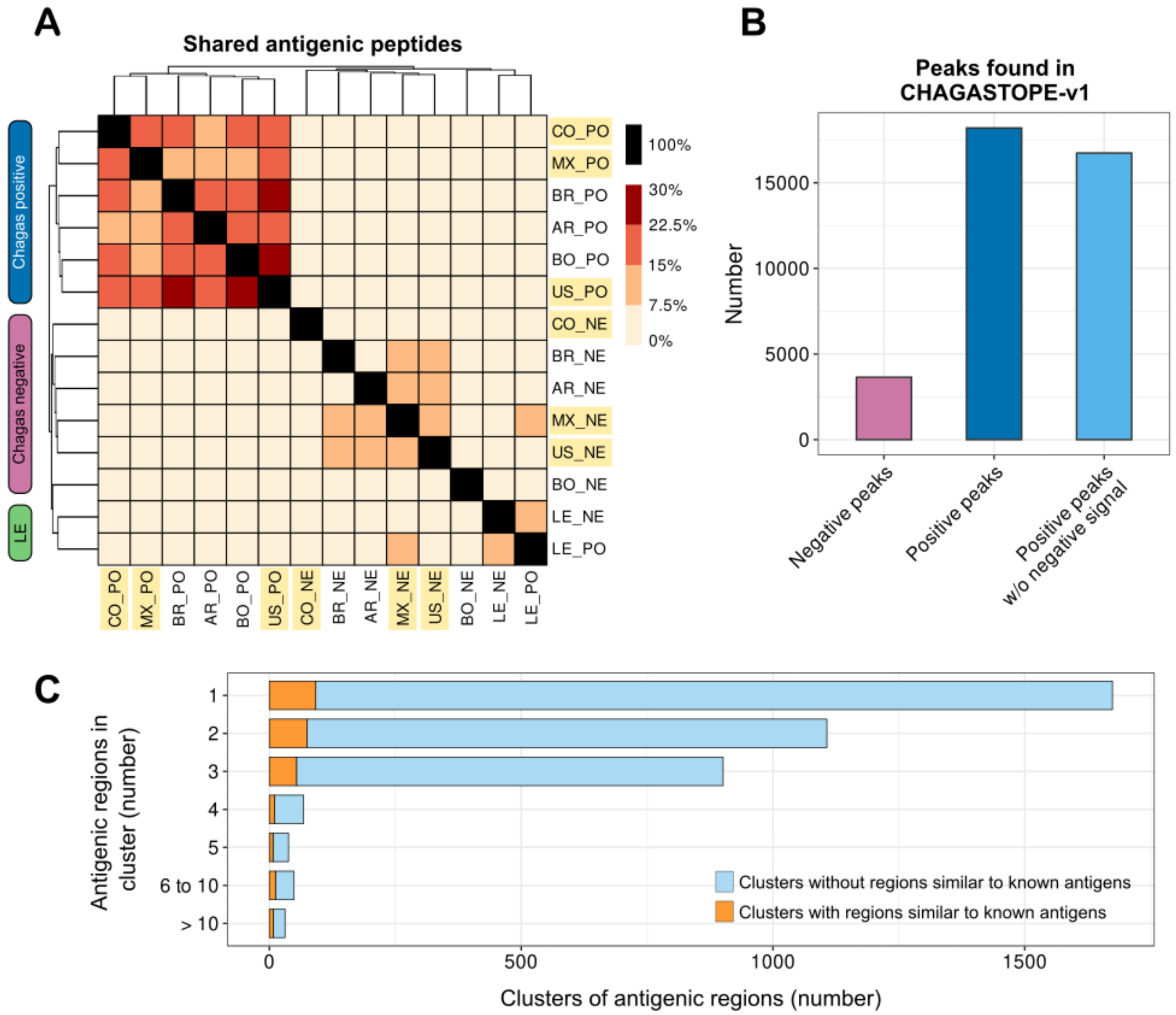

Supplementary Figure S2. Overview of antigenic peptides, peaks and regions in CHAGASTOPE-v1. *Comparative view of the reactivity of pools from Chagas-positive subjects and Chagas-negative (healthy) subjects. See Supplementary Data S2 for the codes of patient serum samples (AR = Argentina, BR = Brazil, BO = Bolivia, CO = Colombia, MX = Mexico, US = United States, LE = Leishmaniasis). Source data for each panel are provided as a Source Data file.* ***A)*** *The heatmap shows the percentage of non-redundant antigenic (positive) peptides that were shared between a pair of serum samples (see Methods). Rows and columns were clustered by similarity, resulting in three distinct clusters as labeled at the left of the plot. The pools from Leishmania-positive and negative subjects were used to analyze cross-reactivity on a later stage (see Supplementary Data S6).* ***B)*** *Summary of the observed peaks in Chagas-positive and Chagas-negative subjects (peaks as per the definition in main text and Methods). The rightmost column shows the number of peaks in Chagas-positive subjects that show no significant signal in the negative samples (see Methods for details).* ***C)*** *Visual summary of how the 9,547 antigenic regions are distributed in the 3,868 non-redundant clusters. To assess if a cluster had regions similar to known antigens, we compared the region's sequences against a set of T. cruzi's linear epitopes found in IEDB (see Methods).*

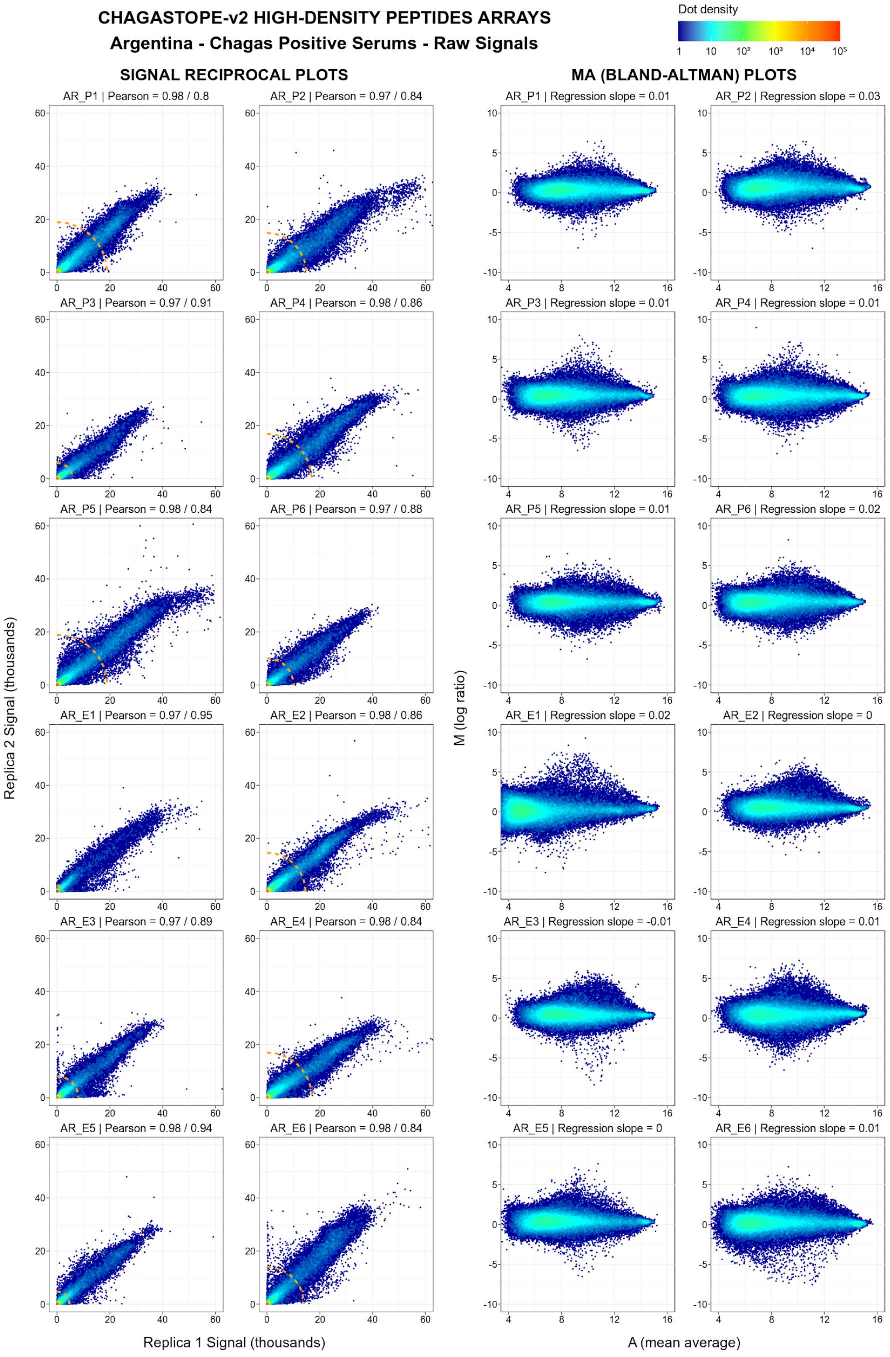

Supplementary Figure S3a. Quality Control of 12-plex microarray assays for AR samples. *Each biological sample (serum sample from a single individual) was assayed in duplicate (technical replicates) in a separate 12-plex slide. The signal correlation between technical replicates is shown both in reciprocal plots and in MA (Bland-Altman) plots. Each point represents one unique peptide or addressable array spot (n = 392,299). The density is shown in a color gradient (see key in figure). In the reciprocal plots, the raw signal data of one replicate is plotted against the second replicate and two Pearson scores are calculated: the first one using all peptides and the second one using only the ones with the top 5% signals, which are those outside the dashed orange line. In MA plots, each signal is replaced by its log2 and then the average signal of the two replicates (A) is plotted against the difference of the two signals (M).*

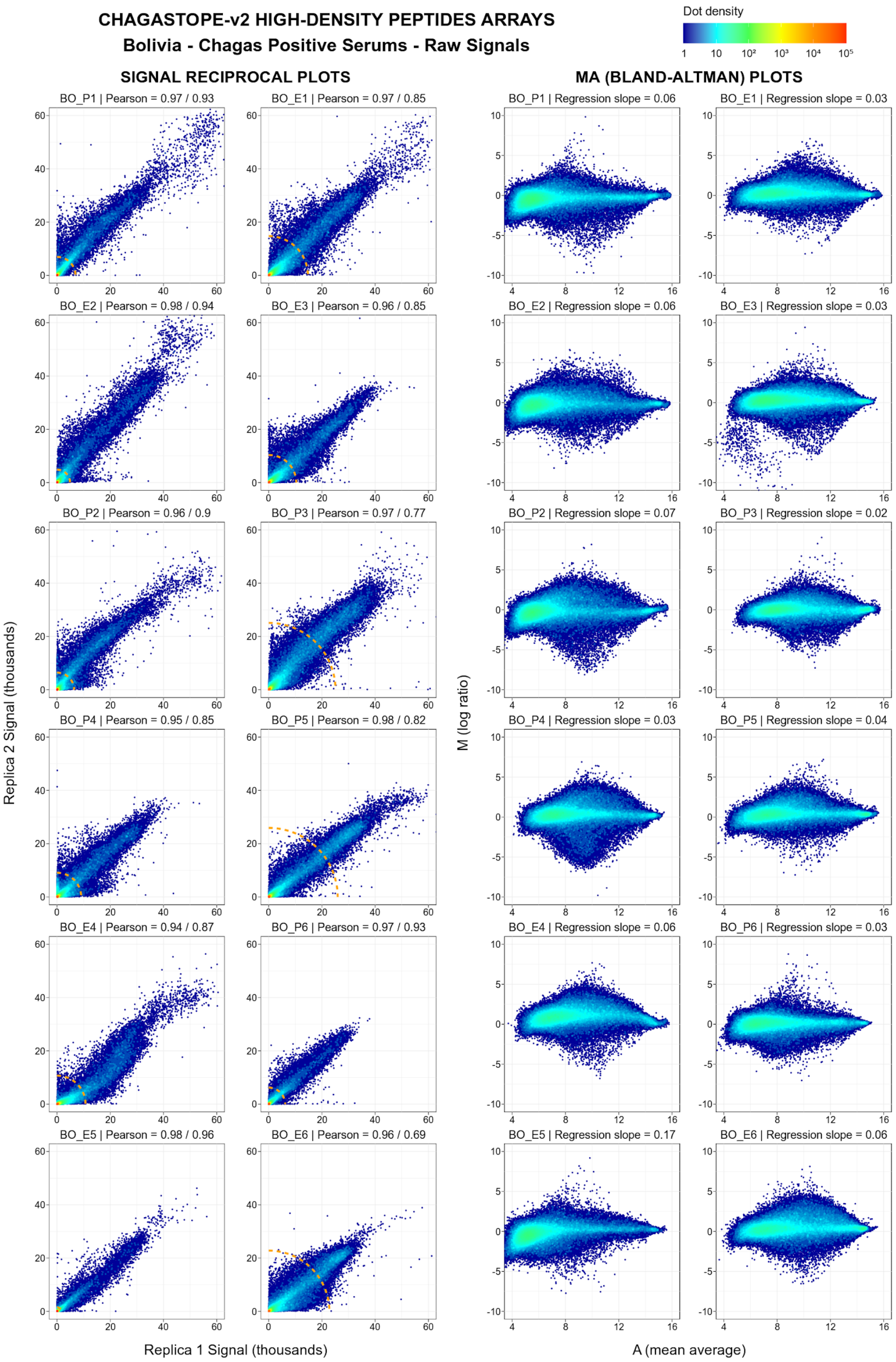

Supplementary Figure S3b. Quality Control of 12-plex microarray assays for BO samples. *The legend is similar to that in Supplementary Figure S3a.*

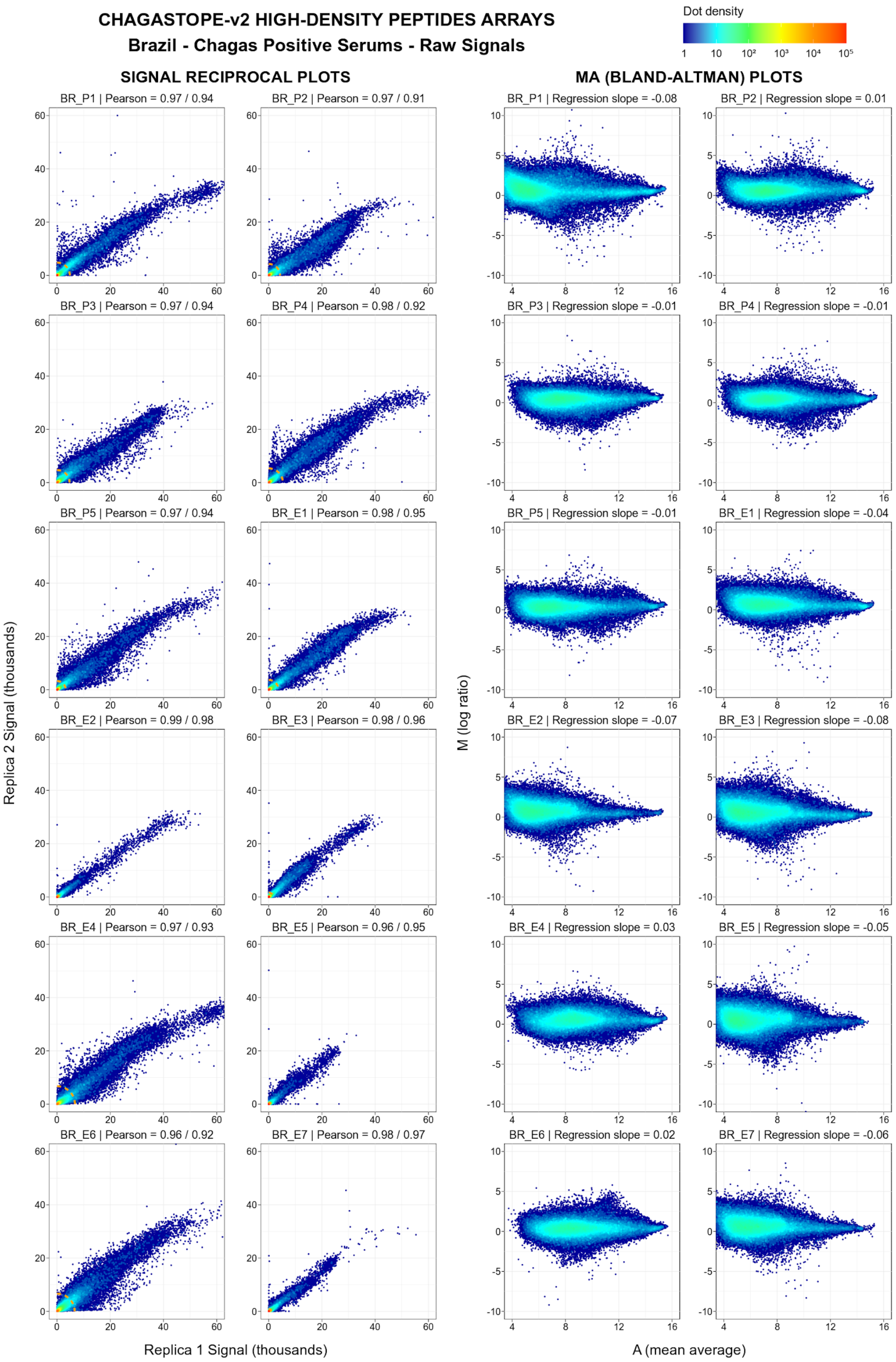

Supplementary Figure S3c. Quality Control of 12-plex microarray assays for BR samples. *The legend is similar to that in Supplementary Figure S3a.*

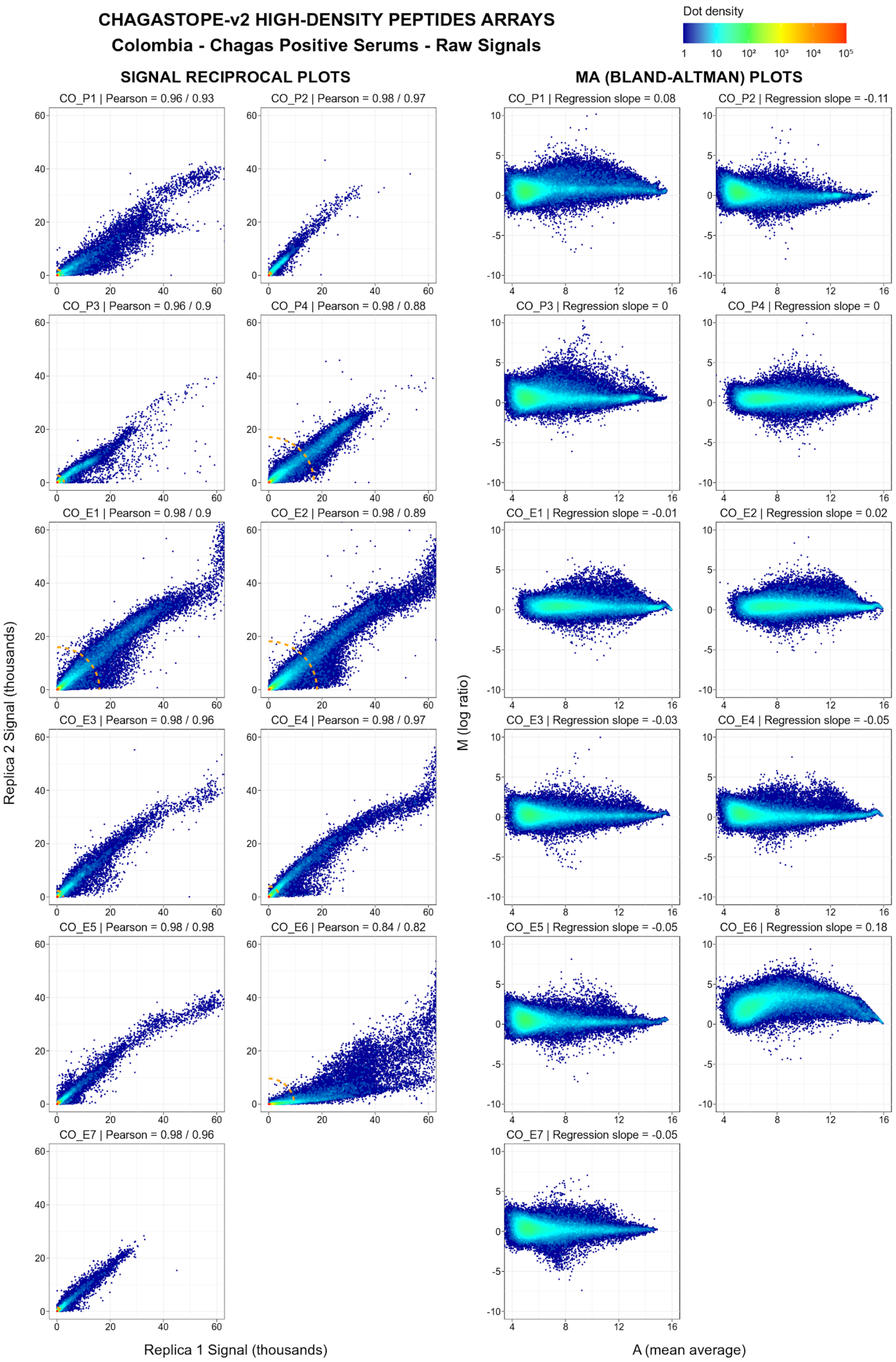

Supplementary Figure S3d. Quality Control of 12-plex microarray assays for CO samples. *The legend is similar to that in Supplementary Figure S3a.*

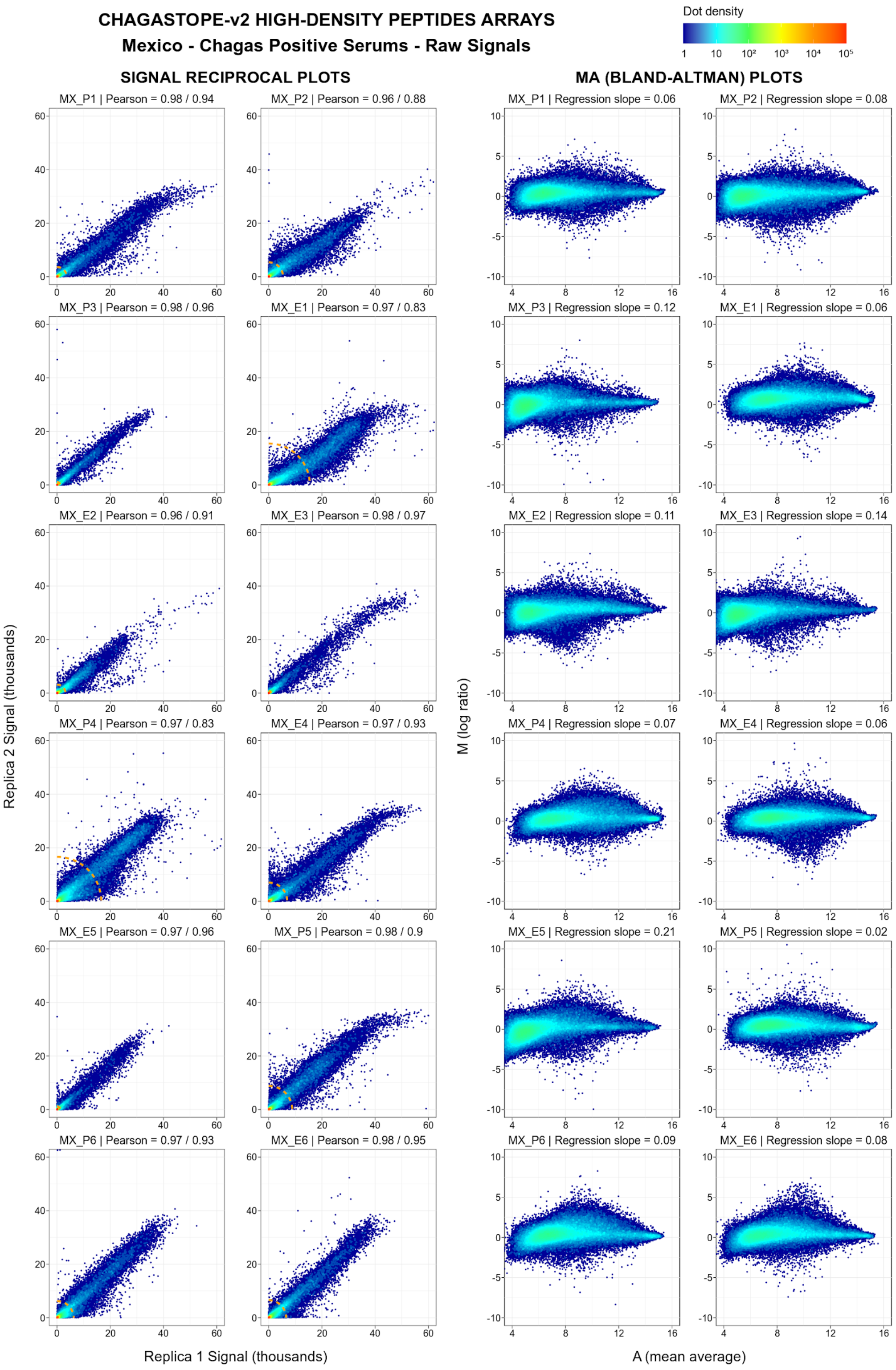

Supplementary Figure S3e. Quality Control of 12-plex microarray assays for MX samples. *The legend is similar to that in Supplementary Figure S3a.*

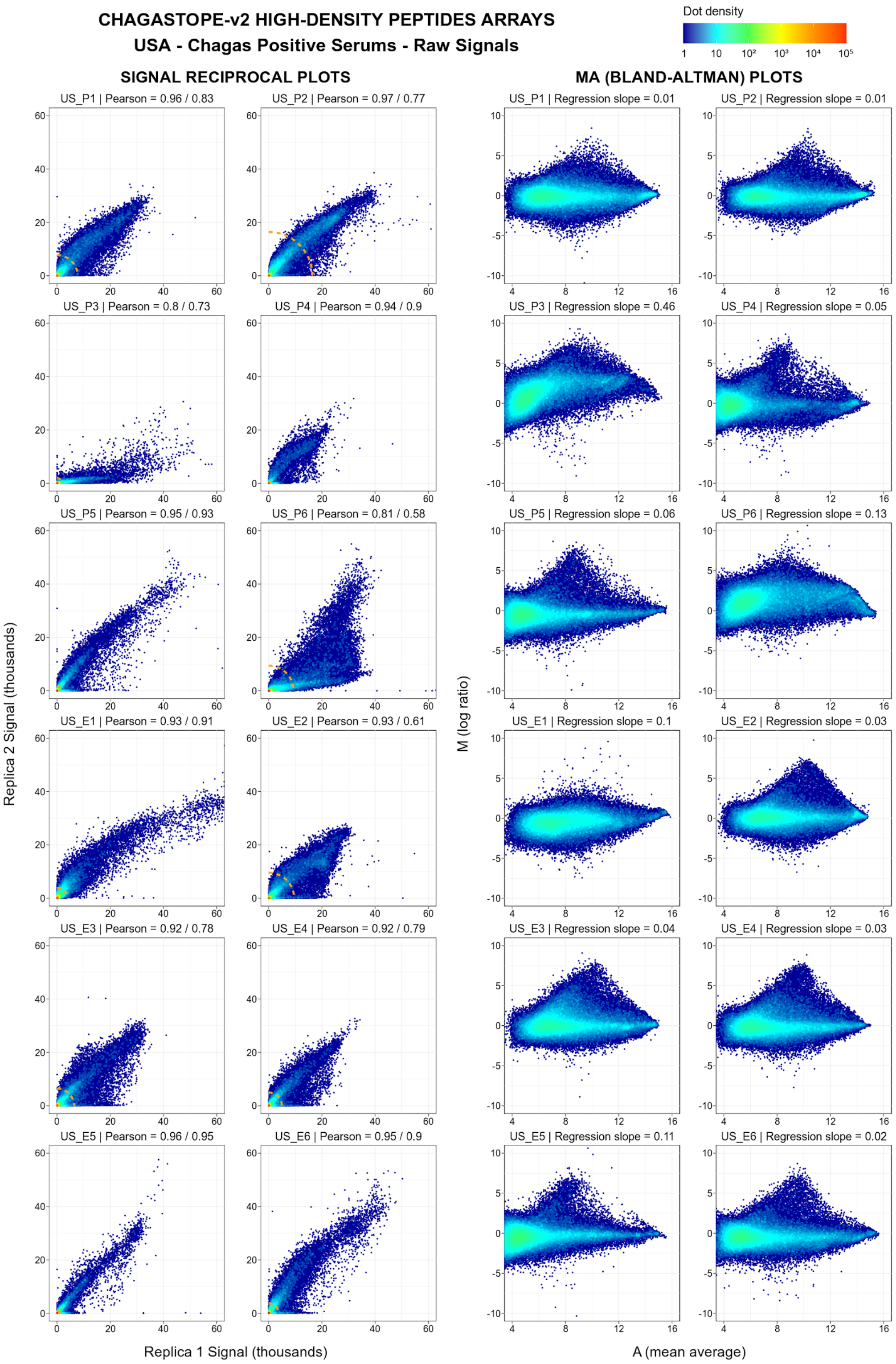

Supplementary Figure S3f. Quality Control of 12-plex microarray assays for the US samples. *The legend is similar to that in Supplementary Figure S3a.*

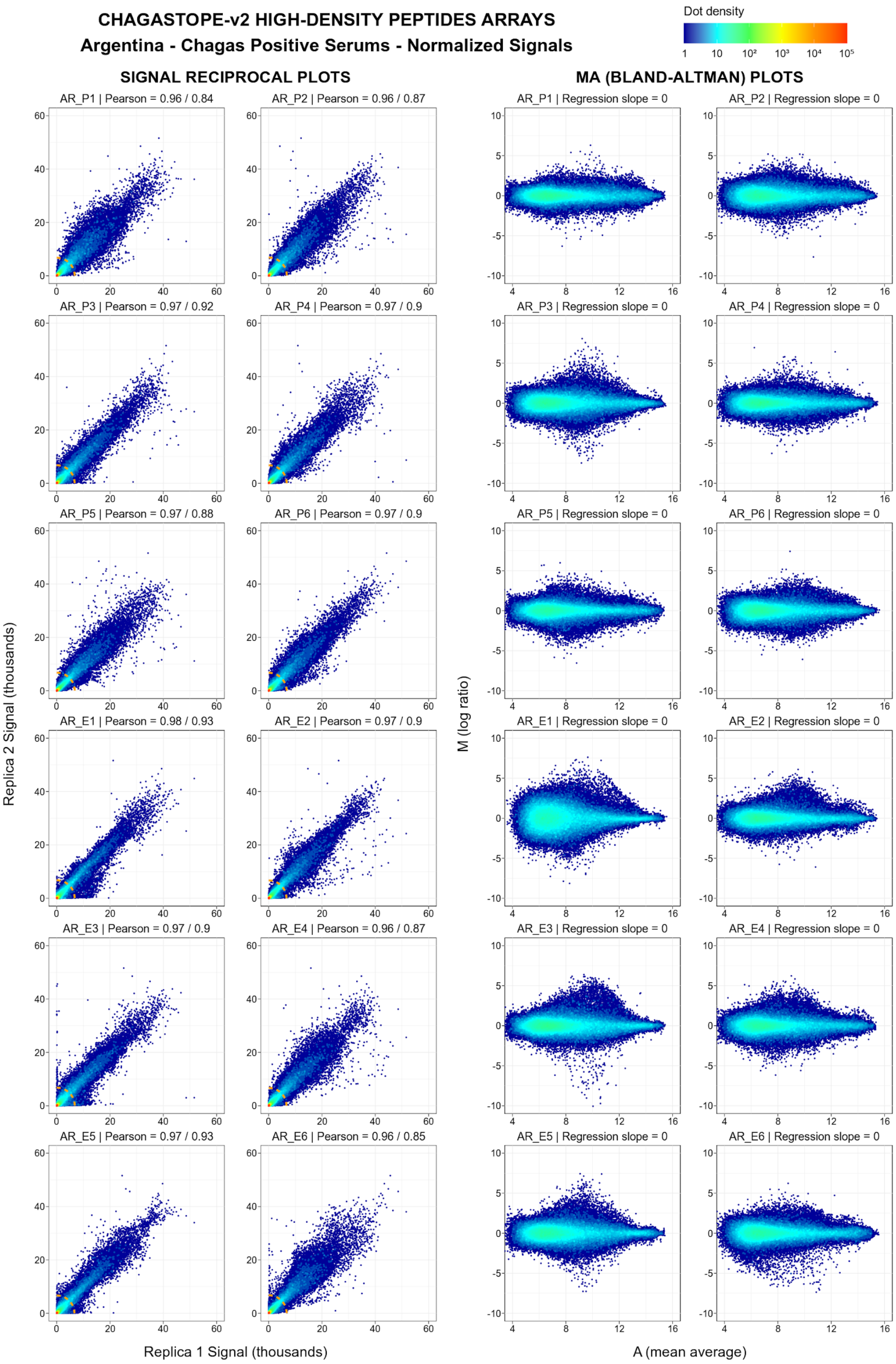

Supplementary Figure S3g. Quality Control of 12-plex microarray assays for AR samples after normalization. *The legend is similar to that in Supplementary Figure S3a, but for the normalised signals.*

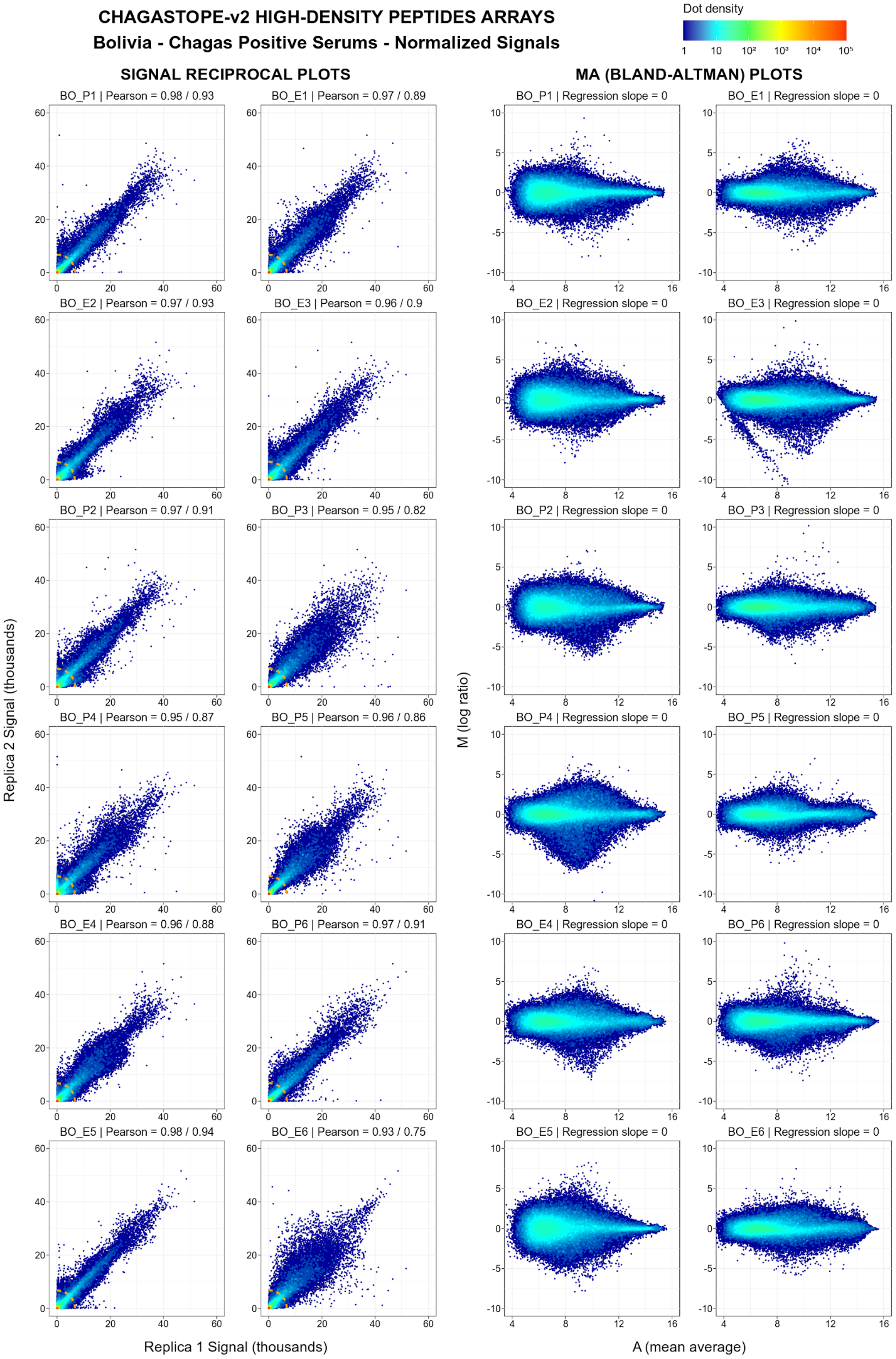

Supplementary Figure S3h. Quality Control of 12-plex microarray assays for BO samples after normalization. *The legend is similar to that in Supplementary Figure S3a, but for the normalised signals.*

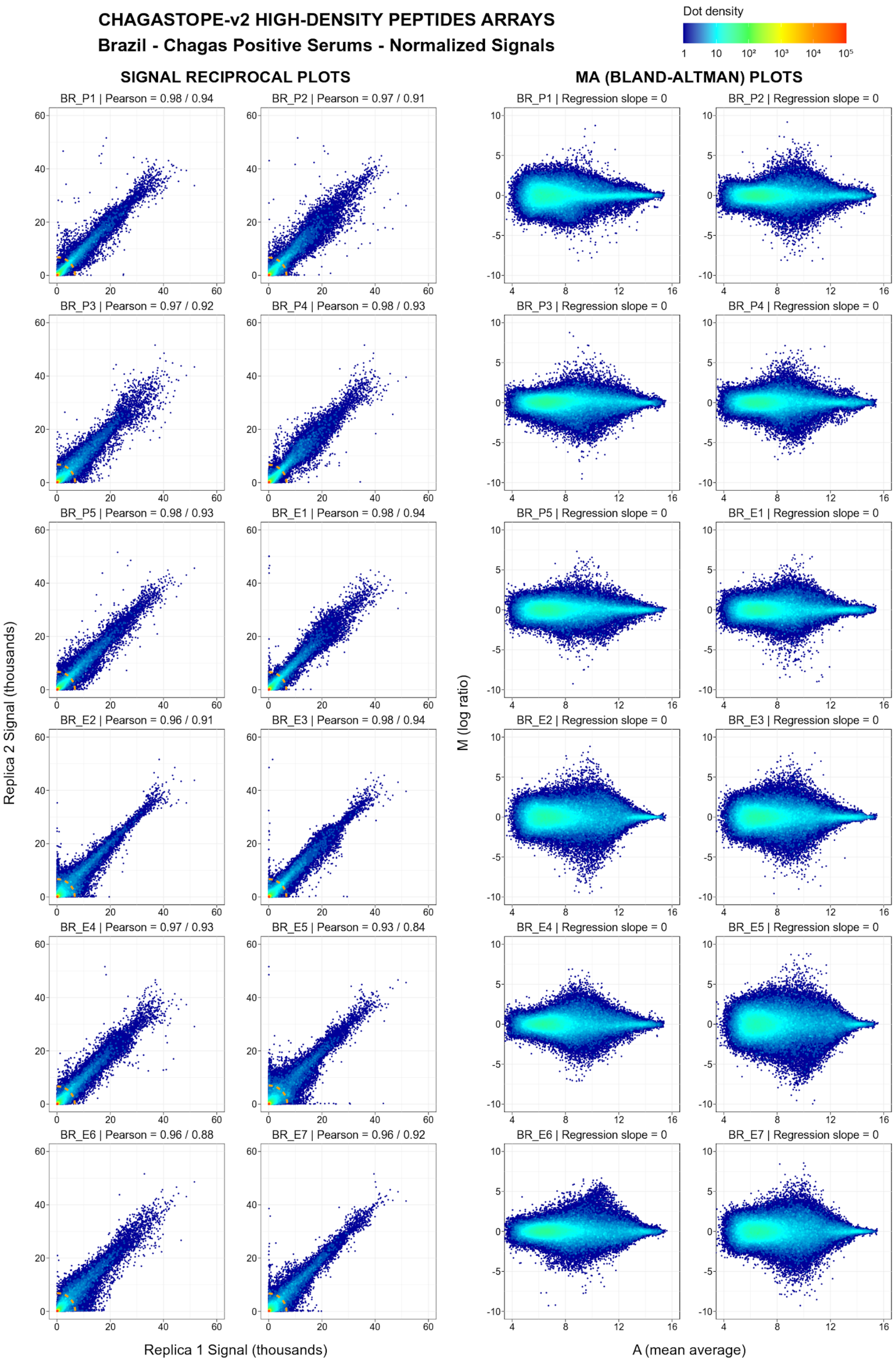

Supplementary Figure S3i. Quality Control of 12-plex microarray assays for BR samples after normalization. *The legend is similar to that in Supplementary Figure S3a, but for the normalised signals.*

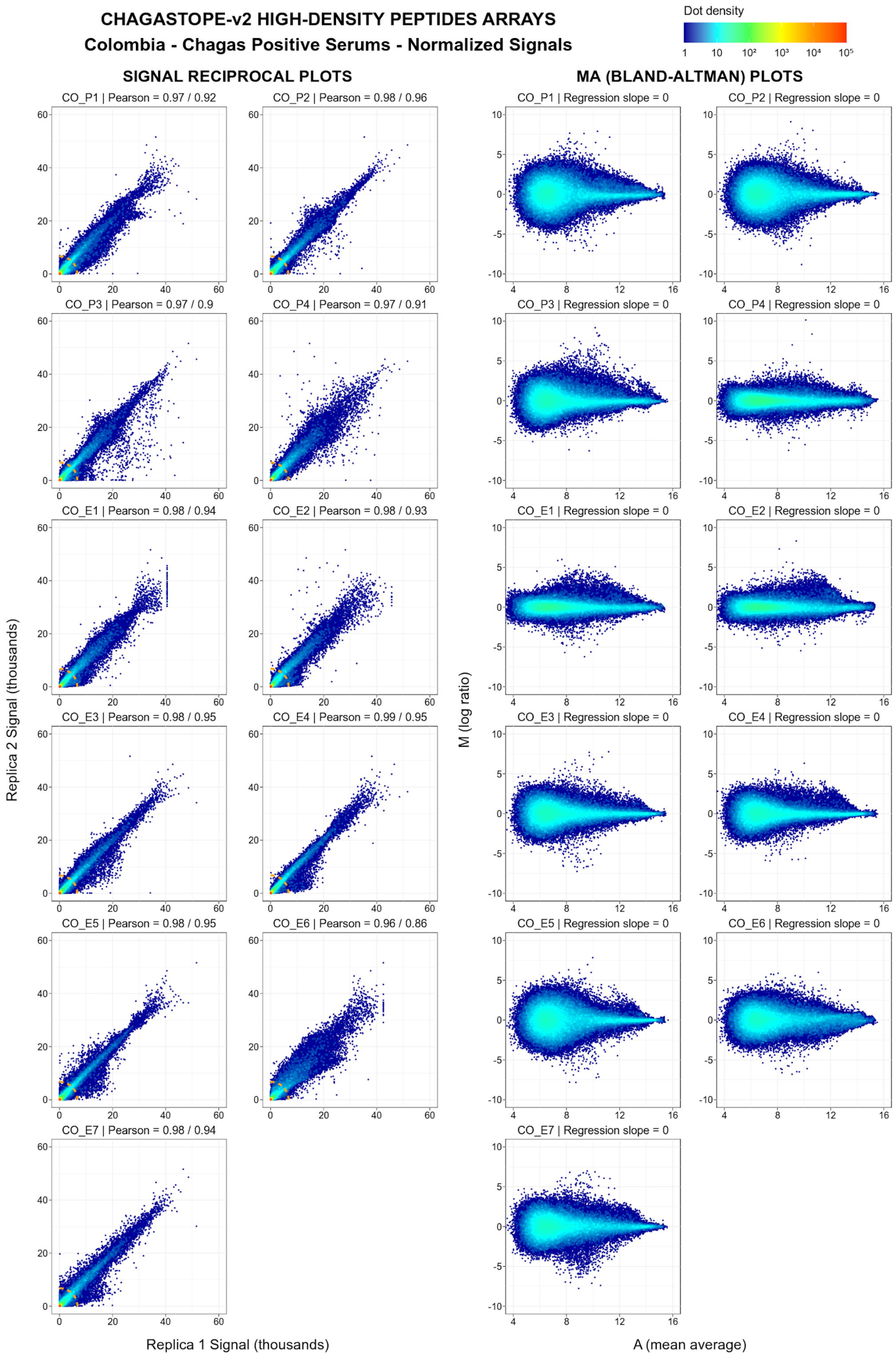

Supplementary Figure S3j. Quality Control of 12-plex microarray assays for CO samples after normalization. *The legend is similar to that in Supplementary Figure S3a, but for the normalised signals.*

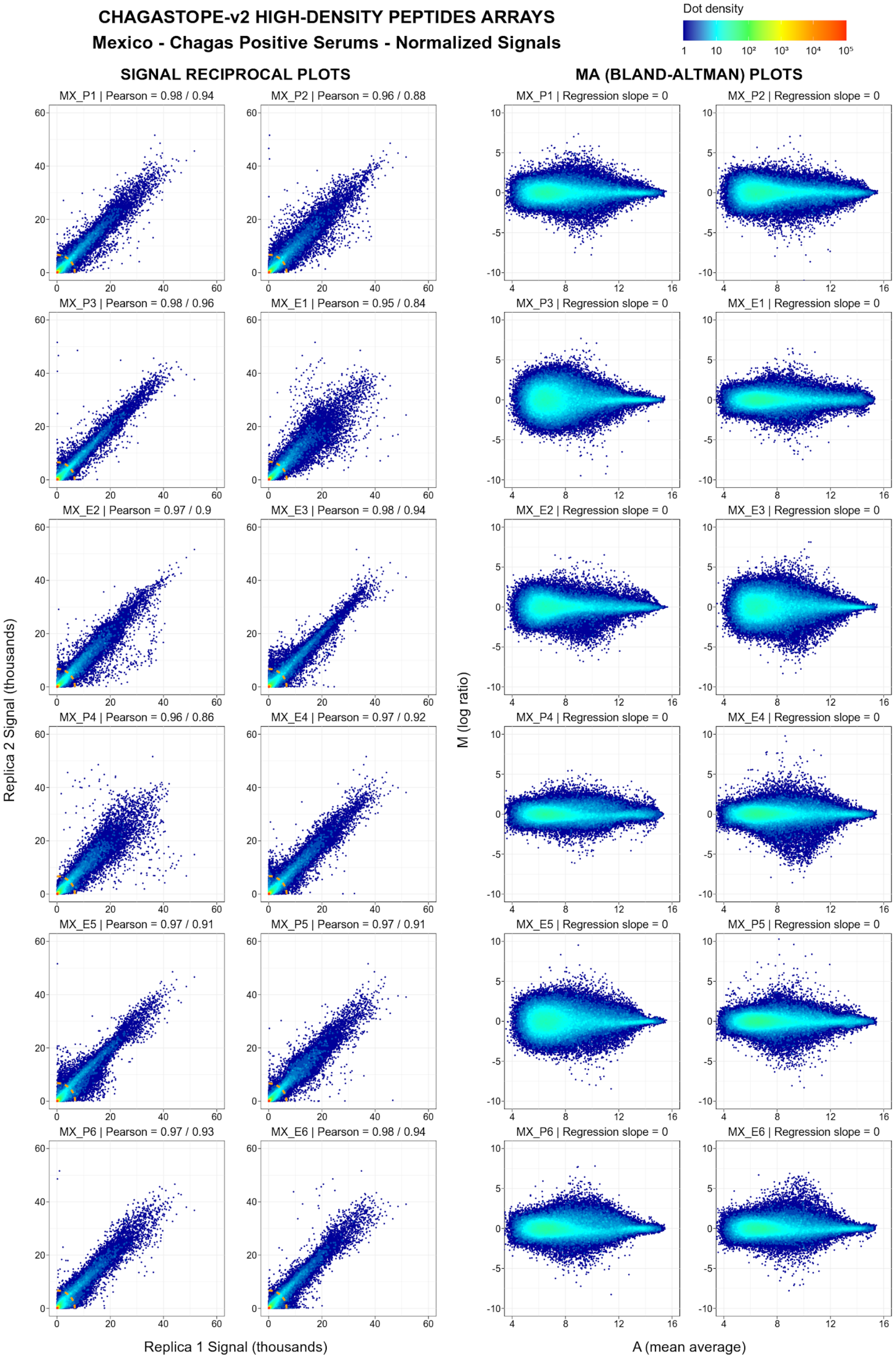

Supplementary Figure S3k. Quality Control of 12-plex microarray assays for MX samples after normalization. *The legend is similar to that in Supplementary Figure S3a, but for the normalised signals.*

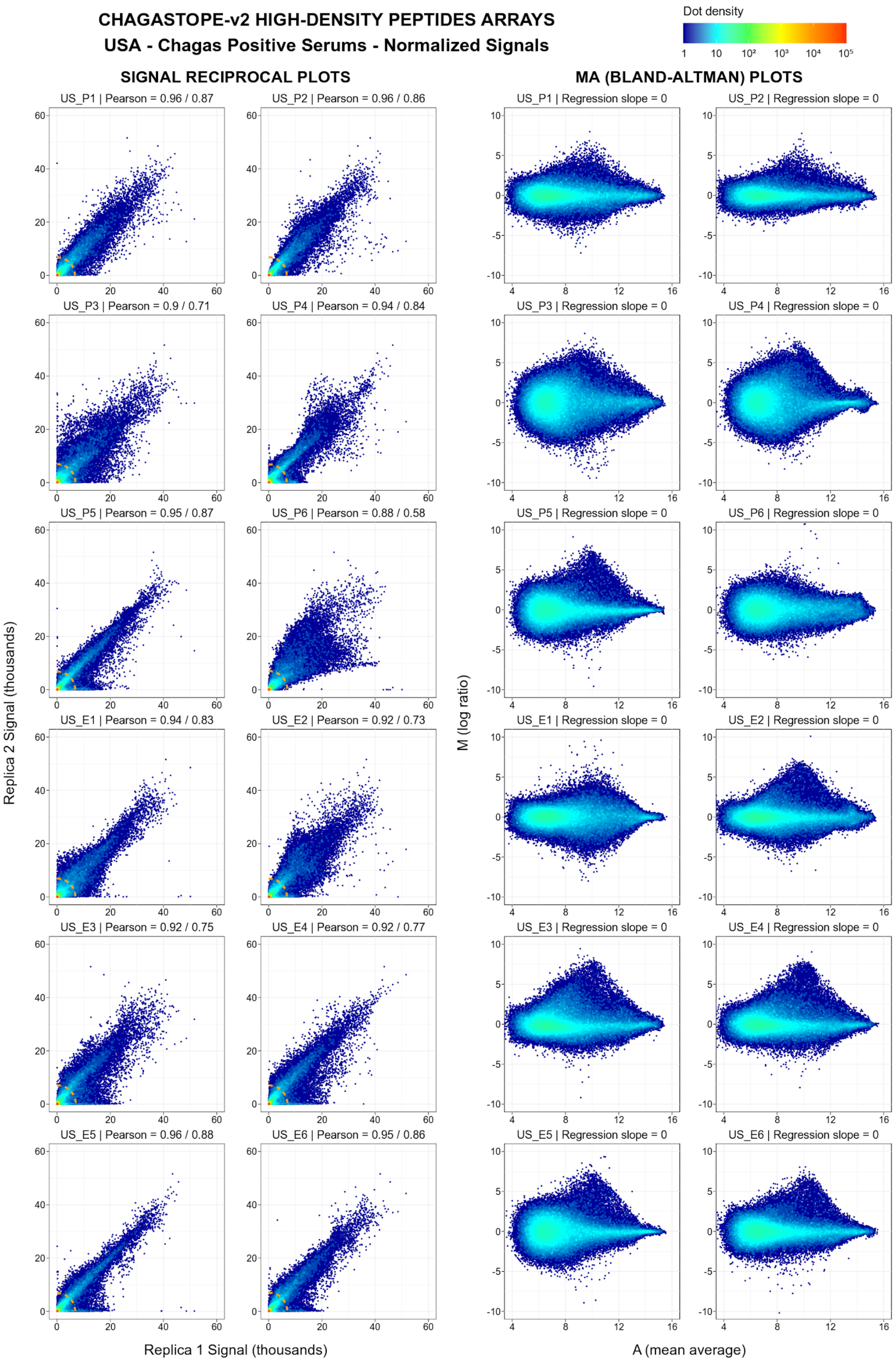

Supplementary Figure S3l. Quality Control of 12-plex microarray assays for the US samples after normalization. *The legend is similar to that in Supplementary Figure S3a, but for the normalised signals.*

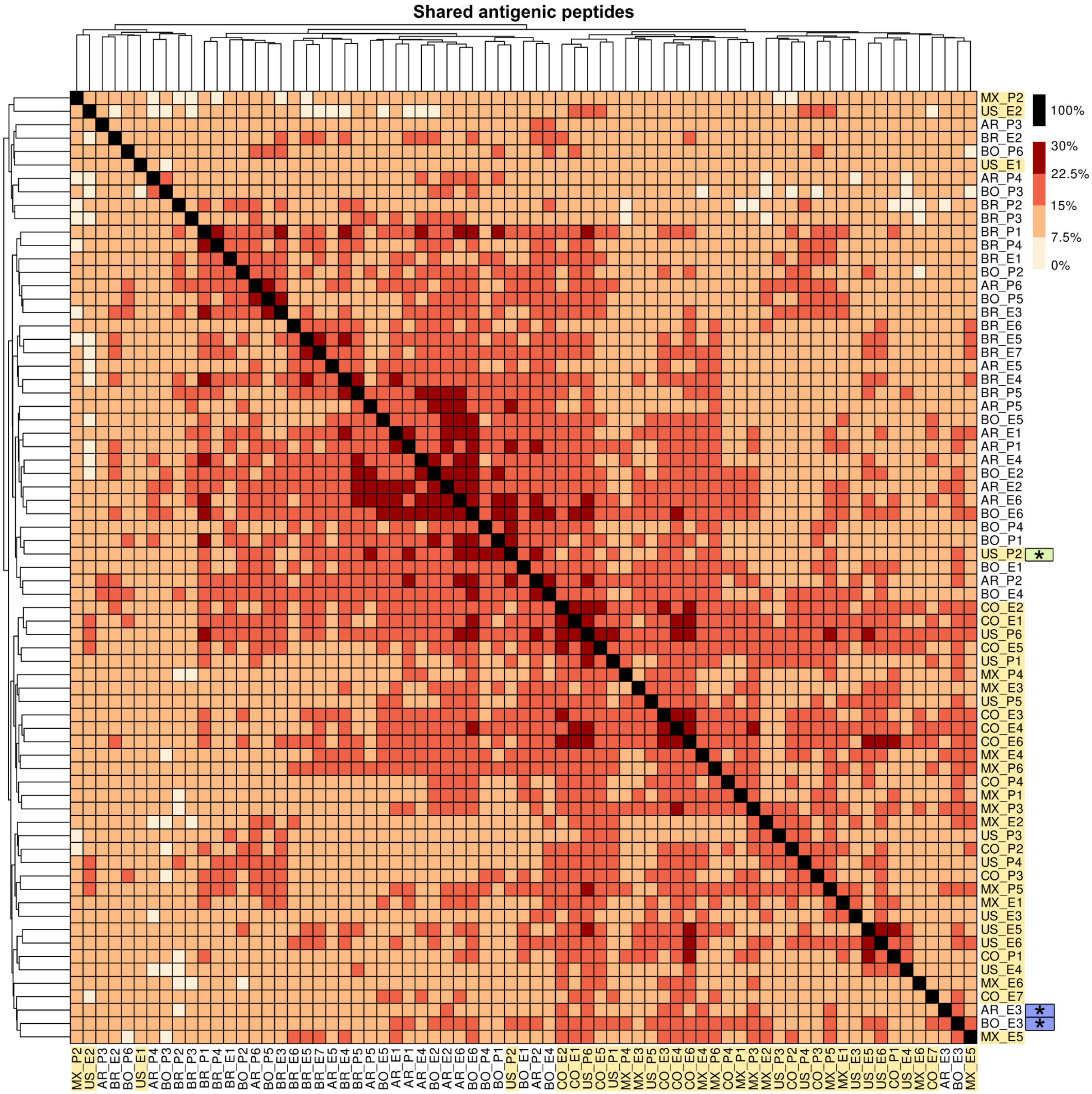

Supplementary Figure S4. Overview of antigenic peptides in CHAGASTOPE-v2. *Comparative view of the reactivity of individual sera from Chagas-positive subjects. The figure is similar to Figure 6c in the main manuscript, except that here sample labels are shown in full. The heatmap shows the percentage of non-redundant antigenic peptides that are shared between a pair of serum samples (see Methods). Rows and columns were clustered by similarity. Samples from North America (Mexico, US) and northern South America (Colombia) are highlighted in yellow, and the asterisks highlight samples that are clustered with sera from other areas (see Figure 6c for details). See Supplementary Data S2 for the codes of patient serum samples (AR = Argentina, BR = Brazil, BO = Bolivia, CO = Colombia, MX = Mexico, US = United States). Source data are provided as a Source Data file.*

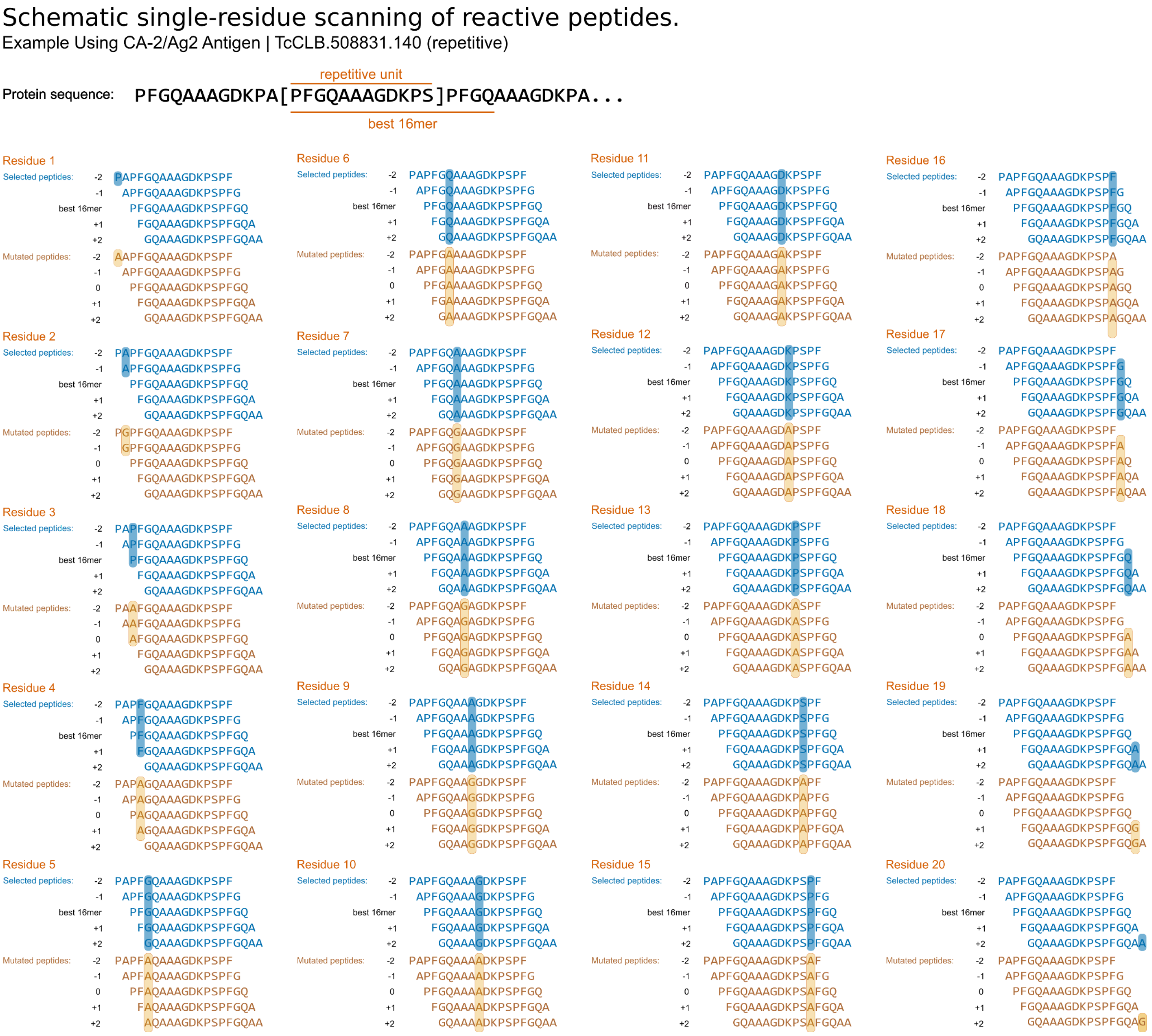

Supplementary Figure S5. Complete visualization of single-residue mutagenesis scanning of epitopes. *Mutational scanning assessment scheme for one analyzed epitope. The same procedure was used to perform single-residue mutagenesis for other epitopes. Antibody-binding (reactivity) was measured for 1 to 5 original (non-mutated) peptide sequences (in blue), and for 1 to 5 mutated peptides per residue position (in orange). In all cases the central peptide (at position 0 in the figure) corresponds to the best 16mer (higher signal) in the epitope. Additional peptides positioned at -2, -1, +1, and +2 were used to measure the effect of mutations in different relative positions within a given epitope. Thus, for any given selected 16mer, a total of 80 mutated peptides were analyzed. Mutations were always to an Alanine, except where the original residue was an Alanine (it was substituted for a Glycine in these cases).*

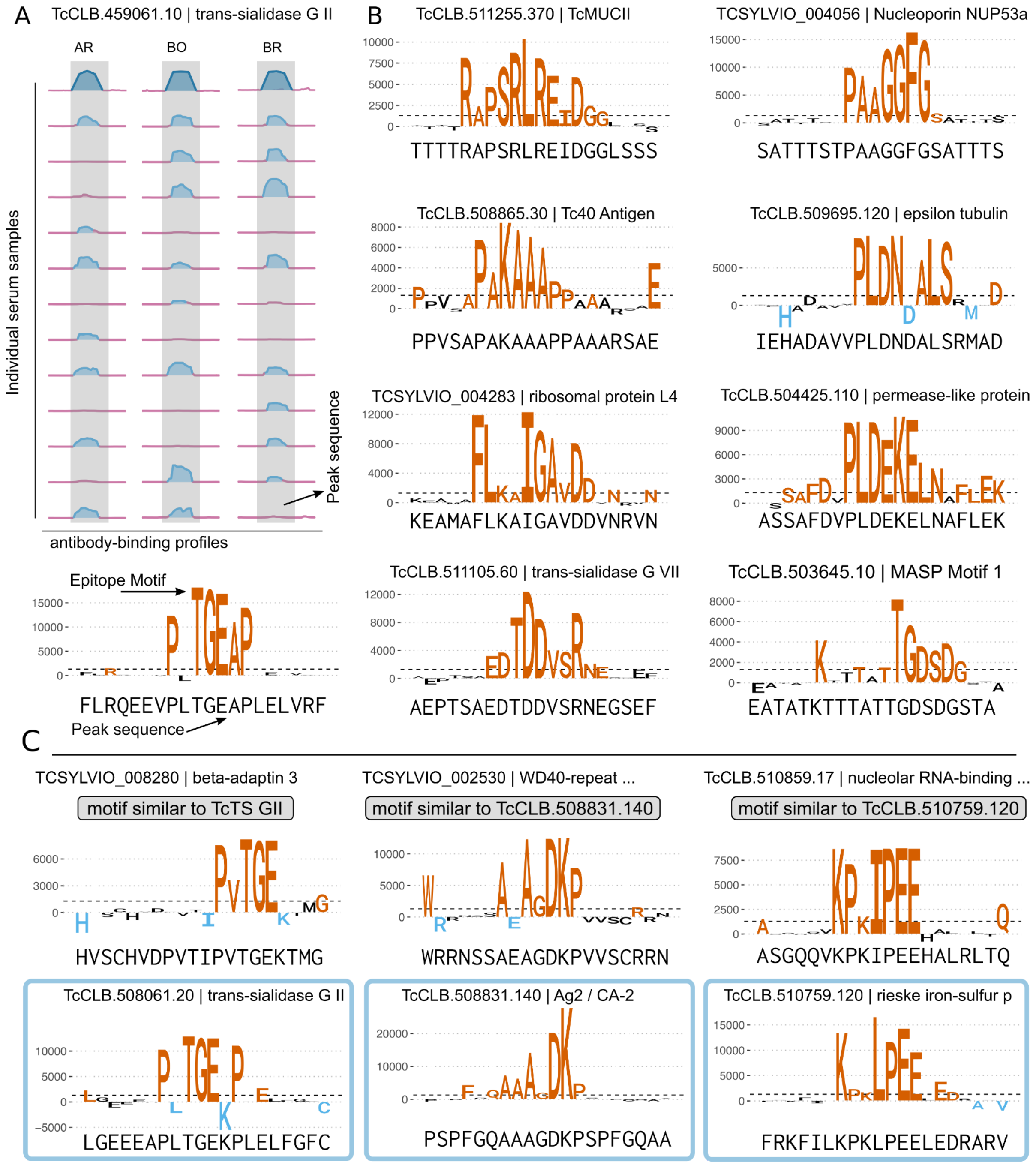

Supplementary Figure S6. A gallery of functional epitope motifs. A) *Antibody-binding profiles for one selected antigen, similar to Figure F_SINGLES (only a subset of 36 of the 71 analyzed sera is shown for clarity). The best 20mer (peak sequence) was subjected to single-residue mutagenesis (see Methods), and the results were summarised as sequence logos.* ***B)*** *Sequence logos showing epitope motifs for additional antigens. Colors for residues that affect antibody-binding are similar as in Figure F_ALASCAN.* ***C)*** *Example cases of antigens with similar core residues (candidate mimotope motifs). Those with higher signal and/or seroprevalence are boxed. Source data for seqlogo plots are provided as a Source Data file.*

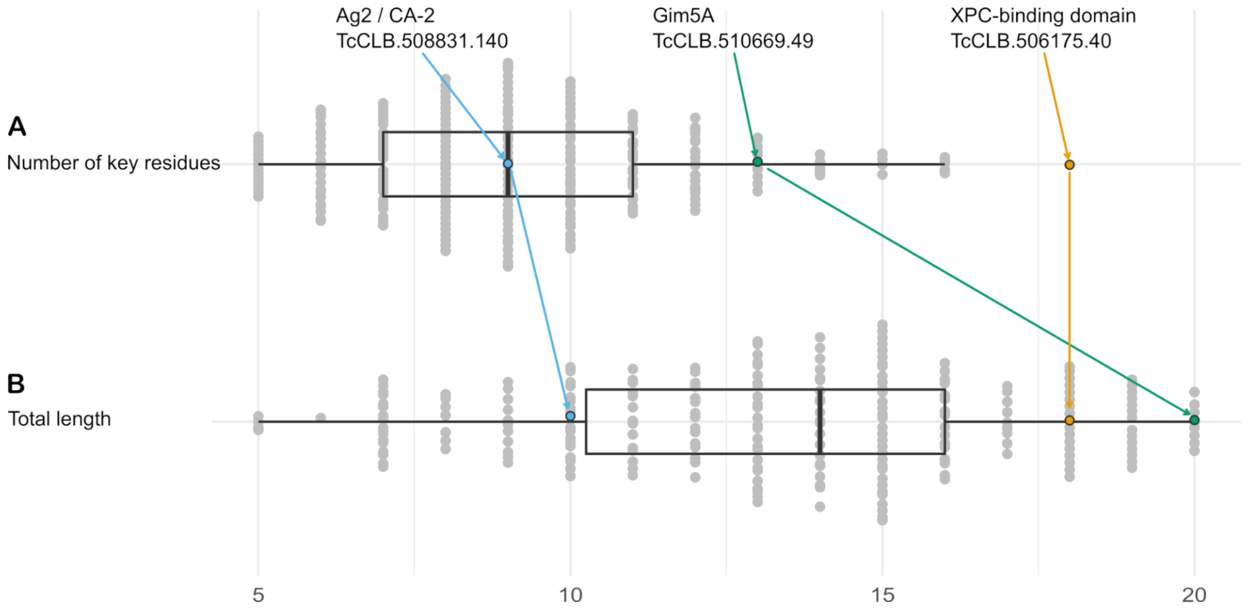

Supplementary Figure S7. Characterization of core functional motifs from single-residue mutational scanning. *Summary of results for the 232 antigenic sequences analyzed with single-residue mutational scanning.* ***A)*** *Distribution of the number of key residues required for antibody binding (see Methods). The boxplot shows the distribution of values, average and most frequent number was 9.* ***B)*** *Distribution of total motif length, where motif length is the span from the first (leftmost) key residue in the motif to the last (rightmost). Average length span was 14, and most frequent length span was 15 residues. Three T. cruzi antigens are mentioned as examples. Source data for each panel are provided as a Source Data file. Boxplots: the upper and lower bounds of the box correspond to the first and third quartiles. Whiskers extend from the box up to 1.5 * IQR (interquartile range) or to the smallest and/or largest value. The centre of the box corresponds to the median value. Source data for these plots are provided as a Source Data file.*

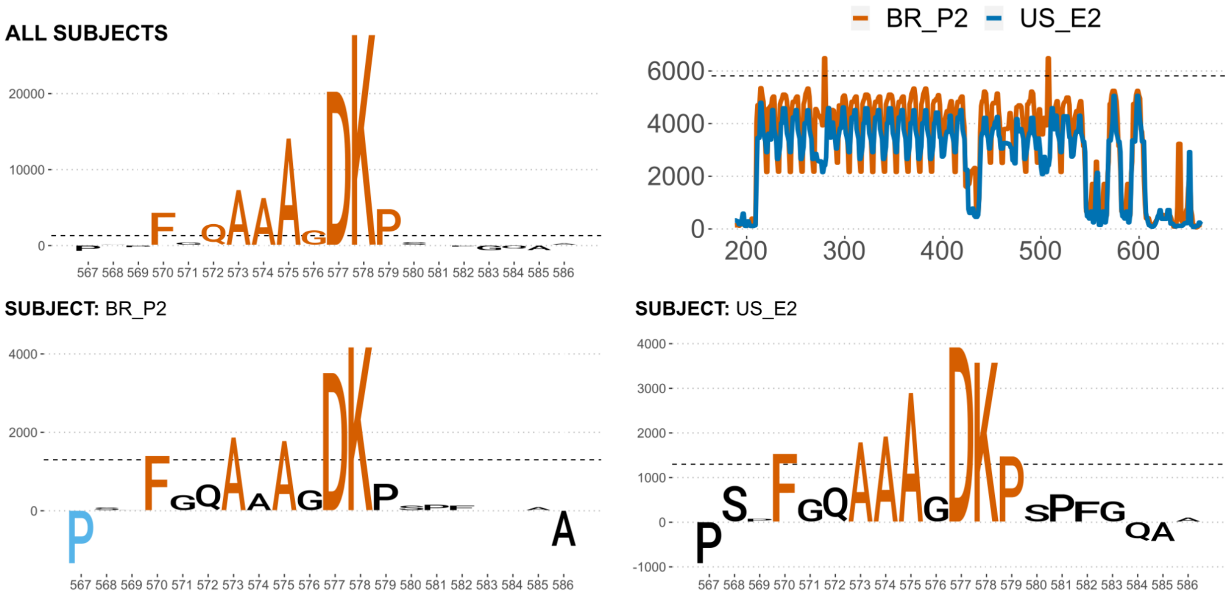

Supplementary Figure S8. Single-residue mutational scanning of the Ag2/CA-2 antigen for subjects that were negative or displayed low signal against this epitope. *Top left, sequence logo summarizing key residues revealed by mutational scanning (derived from analysis of all subjects, logo is the same as in Figure F_ALASCAN in the main manuscript). Top right, antibody-binding profile for the BR_P2 and US_E2 subjects and signal threshold (dashed line). Bottom, sequence logos of two subjects that display low signal and/or are negative with the chosen signal threshold. Source data for signal and seqlogo plots are provided as a Source Data file.*

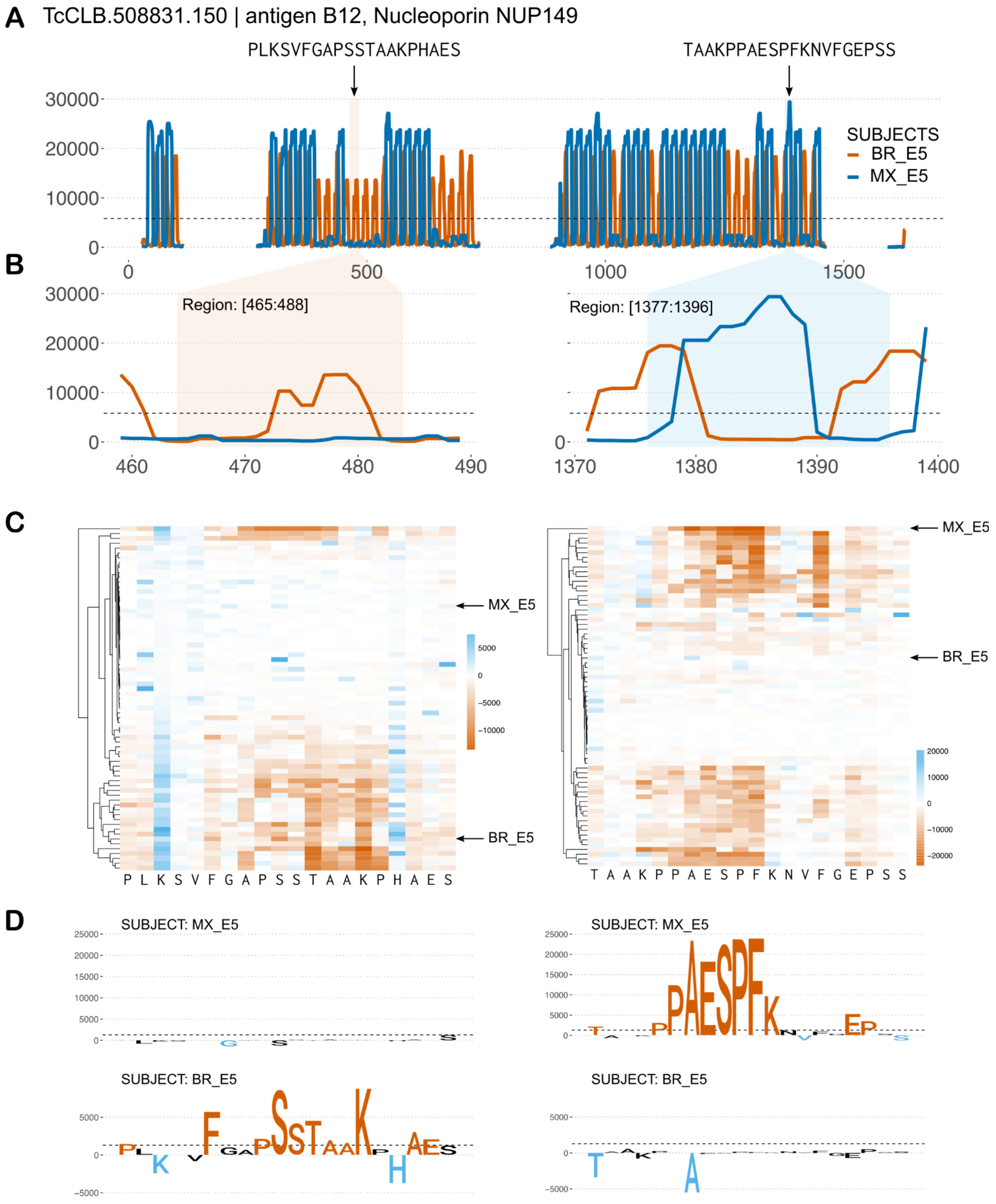

Supplementary Figure S9. Single-residue mutational scanning for antigen B12 (TcCLB.508831.150). *Two sequences from the repetitive antigenic region of the B12 antigen [1] were subjected to mutational scanning as described (see main text).* ***A)*** *Individual antibody-binding signal profiles for two subjects; MX_E5 (blue) and BR_E5 (orange). The X axis represents the middle position of the peptide in the sequence of the protein. The Y axis represents the normalised signal (see methods for details) for each individual; regions without data were not present in the CHAGASTOPE-V2 design.* ***B)*** *Zoom in regions [458:490] (left) and [1370:1400] (right).* ***C)*** *Heatmaps summarizing the mutational scanning for all residues and all subjects (see legend of Figure F_ALASCAN).* ***D)*** *Sequence logos summarizing data for individual cases as denoted (y-axis: signal change in arbitrary units). Colors follow heatmap.*

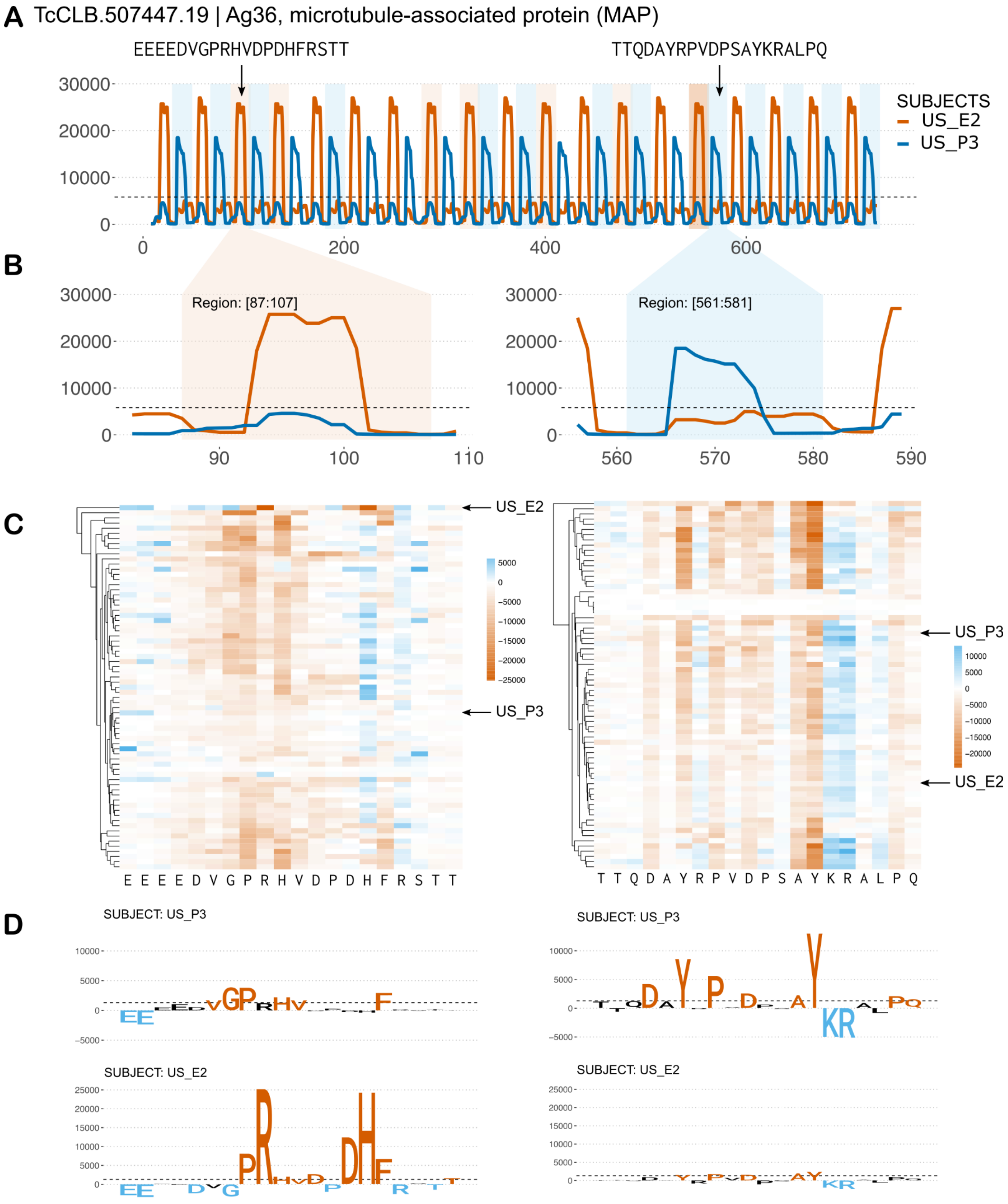

Supplementary Figure S10. Single-residue mutational scanning for antigen Ag36 (TcCLB.507447.19). *Two sequences from the repetitive region of the Ag36 antigen [2,3] were subjected to mutational scanning as described (see main text). The legend is similar to that in Supplementary Figure S9. Subjects: US_P3 (blue) and US_E2 (orange) and zoom in regions [82:110] (left) and [555:590] (right).*

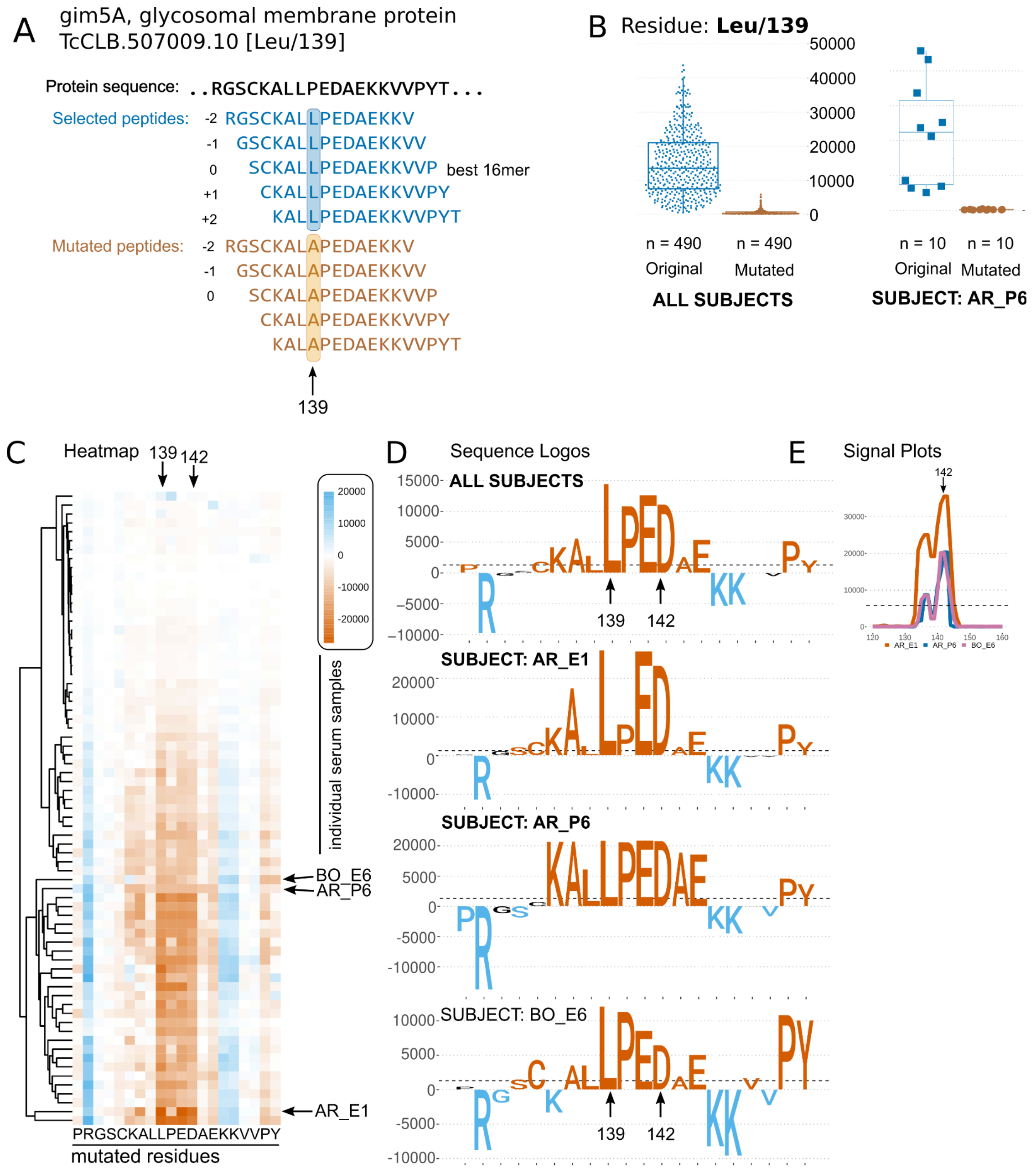

Supplementary Figure S11. Single-residue mutational scanning of the novel Gim5A antigen. *The single antigenic peak in the Gim5A protein was subjected to mutational scanning as described (see main text). Figure elements as in Fig7 (main manuscript).* ***A)*** *Schematic representation of the mutational scanning procedure, shown for one residue only (Leu139), for clarity.* ***B)*** *Average signal of the original and mutated peptides for the Leu/139 residue for all positive sera (n = 43 independent biological samples; n = 245 peptides assayed in duplicate) (left) and for one selected subject (n = 1 biological sample; n = 5 peptides assayed in duplicate) (right). Boxplots: the upper and lower bounds of the box correspond to the first and third quartiles. Whiskers extend from the box up to 1.5 * IQR (interquartile range) or to the smallest and/or largest value. The centre of the box corresponds to the median value.* ***C)*** *Heatmap summarising the mutational scanning for all residues and all subjects.* ***D)*** *Sequence logos summarising data for all positive sera (top), or for individual cases as denoted.* ***E)*** *Antibody binding signal plots for the selected subjects.*

Tandemly repeated antigens are known important targets for the host immune response, and there is a long track record of studies on these types of antigens in *T. cruzi* [3–5]. Many of these are present in commercial diagnostic tests [6]. Single-residue mutational scanning revealed key residues that are necessary for antibody binding. In Supplementary Figure S12 we show an example of a repetitive antigen [7] where the key residues identified by mutagenesis (sequence logo in Figure S12**A**) are shown alongside the peptide with the highest signal in the arrays (Figure S12**B**), and the tandem repeats identified by XSTREAM [8] in Figure S12**C**. The same procedure and visualisation has been produced for 18 other antigens carrying tandemly repeated epitopes (included in Supplementary File S7).

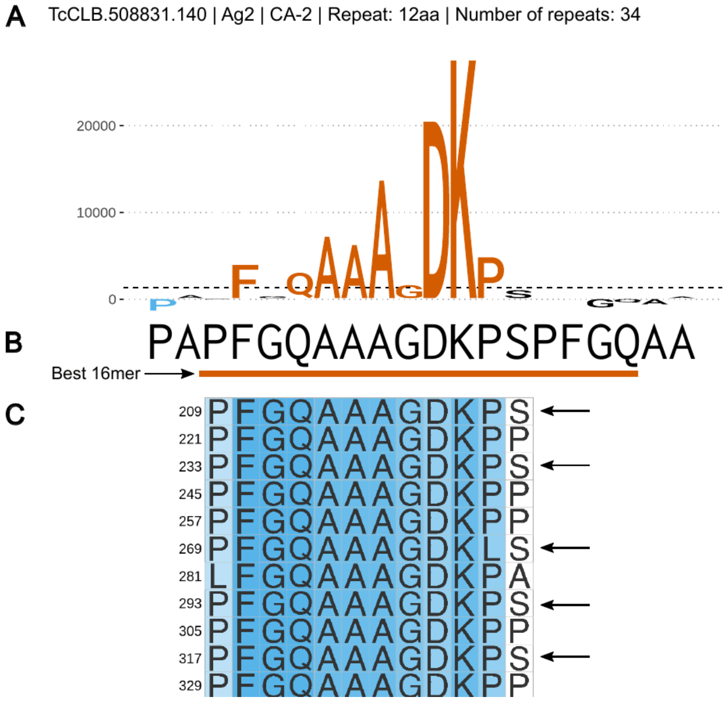

Supplementary Figure S12. Single-residue mutational scanning of a tandemly repeated epitope. *The repetitive antigenic sequence of antigen Ag2/CA-2 was subjected to a mutational scanning as described (see main text). Here, we presented it aligned with the tandem repeated sequence identified by Xstream software.* ***A)*** *Sequence logo showing epitope motifs. Colors for residues that affect antibody-binding are similar as in Figure F_ALASCAN.* ***B)*** *Sequence studied in single-residue mutational scan.* ***C)*** *Tandem repeats sequence identified by Xstream software, only first 11 repeats are shown, see Supplementary File S7 for the complete list. The peptide identified in this study as the most antigenic for this protein is highlighted; perfect repetitions of this sequence are indicated with arrows.*

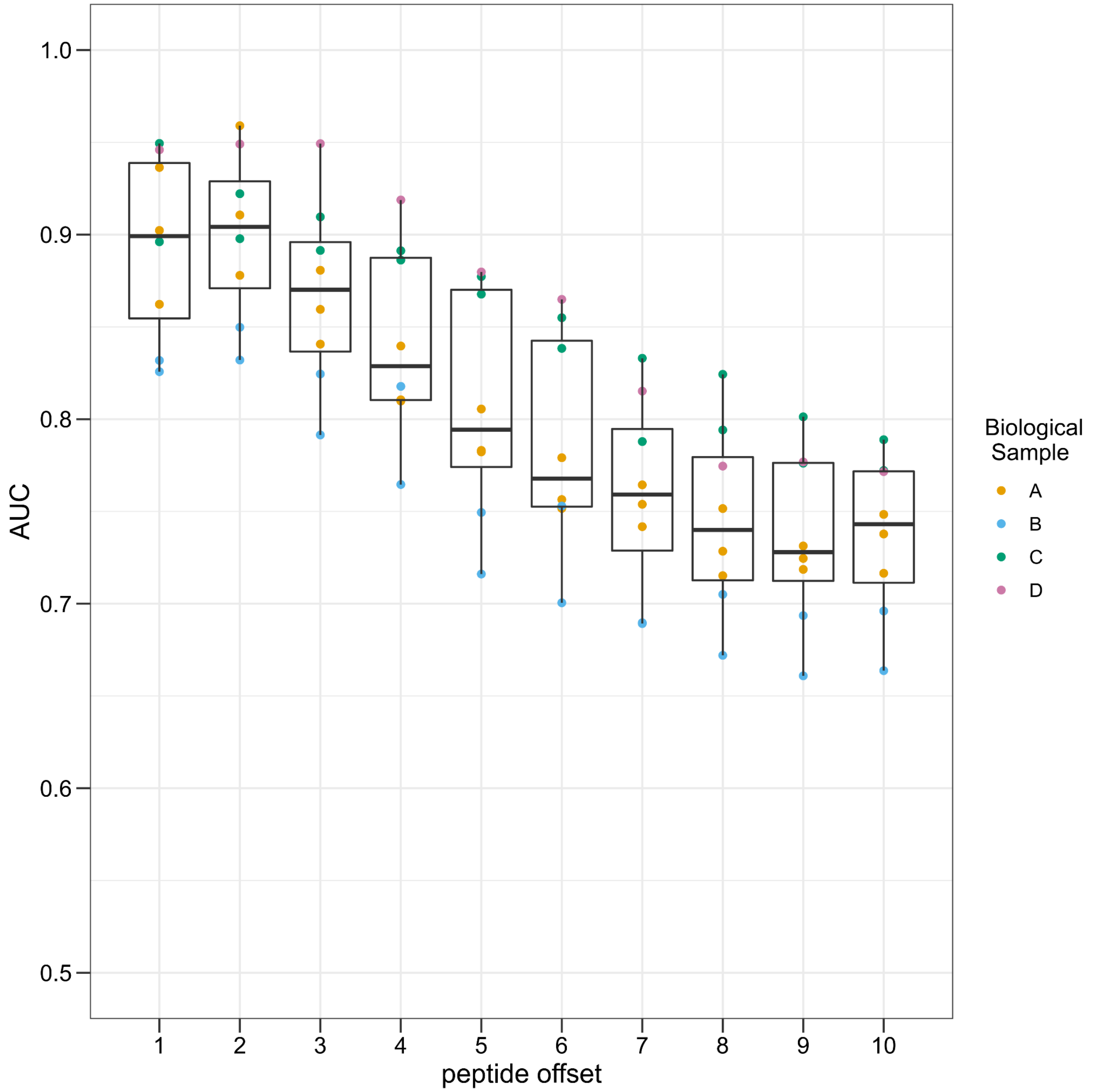

Supplementary Figure S13. Performance at the task of mapping known linear epitopes using tiling peptide strategies. *Data from high-resolution peptide arrays (n=8 array experiments) (Carmona et al 2015) were used to simulate different offsets. The original data contained maximal resolution epitope mapping assays (sliding window = 15 residues; offset = 1 residue). The simulated data was produced by skipping a different number of consecutive peptides in the sequence to produce lower resolution epitope mapping scenarios (skipping every other peptide produces offset = 2; skipping two consecutive peptides produces offset = 3, etc). In each simulation, we calculated the Area Under the ROC Curve (AUC) to measure the performance of a given offset at mapping the epitopes of known antigens. Boxplots: the upper and lower bounds of the box correspond to the first and third quartiles. Whiskers extend from the box up to 1.5 * IQR (interquartile range) or to the smallest and/or largest value. The centre of the box corresponds to the median value. Each point represents an independent microarray assay. Points with the same color represent technical replicates (assays with the same biological sample). Source data for this plot are provided as a Source Data file.*

TCSYLVIO_004056 (MX_P1)
--PAAGGFGS--

PEPTIDE SEQ	RAW SIGNAL
GFGSATTTSTPAAGGF	1,263 ± 148
FGSATTTSTPAAGGFG	2,243 ± 419
GSATTTSTPAAGGFGS	23,620 ± 4,471
SATTTSTPAAGGFGSA	25,405 ± 6,031
ATTTSTPAAGGFGSAT	26,096 ± 4,072
TTTSTPAAGGFGSATT	25,753 ± 6,515
TTSTPAAGGFGSATTT	26,565 ± 9,190
TSTPAAGGFGSATTTS	24,911 ± 9,337
STPAAGGFGSATTTSA	23,640 ± 6,389
TPAAGGFGSATTTSAP	23,415 ± 7,749
PAAGGFGSATTTSAPA	18,012 ± 8,493
AAGGFGSATTTSAPAV	2,357 ± 1,085
AGGFGSATTTSAPAVG	804 ± 410

TcCLB.511517.37 (US_E6)
--TSDDIHE--

PEPTIDE SEQ	RAW SIGNAL
VLLGFTNRWHLTSDDI	31 ± 17
LLGFTNRWHLTSDDIH	14,222 ± 6,018
LGFTNRWHLTSDDIHE	19,955 ± 4,099
GFTNRWHLTSDDIHEA	20,136 ± 1,543
FTNRWHLTSDDIHEAV	22,578 ± 3,170
TNRWHLTSDDIHEAVN	20,974 ± 3,782
NRWHLTSDDIHEAVNR	20,944 ± 4,410
RWHLTSDDIHEAVNRL	17,475 ± 3,769
WHLTSDDIHEAVNRLV	10,061 ± 4,054
HLTSDDIHEAVNRLVD	14,760 ± 4,950
LTSDDIHEAVNRLVDC	23,361 ± 3,844
TSDDIHEAVNRLVDCA	16,623 ± 4,153
SDDIHEAVNRLVDCAT	420 ± 356
DDIHEAVNRLVDCATP	44 ± 20

TCSYLVIO_002216 (AR_E1)
--SGVEDK--P--

PEPTIDE SEQ	RAW SIGNAL
PQDTQGLQGGVSGVED	28 ± 4
QDTQGLQGGVSGVEDK	23,943 ± 2,959
DTQGLQGGVSGVEDKL	25,533 ± 4,399
TQGLQGGVSGVEDKLL	28,238 ± 5,617
QGLQGGVSGVEDKLLP	28,339 ± 3,782
GLQGGVSGVEDKLLPA	27,367 ± 5,654
LQGGVSGVEDKLLPAS	32,049 ± 5,386
QGGVSGVEDKLLPASS	28,613 ± 5,921
GGVSGVEDKLLPASSR	28,226 ± 4,813
GVSGVEDKLLPASSRP	31,642 ± 6,316
VSGVEDKLLPASSRPL	33,752 ± 7,394
SGVEDKLLPASSRPLE	18,231 ± 4,331
GVEDKLLPASSRPLEE	1,370 ± 477
VEDKLLPASSRPLEEE	36 ± 13

TcCLB.508319.30 (BR_E1)
--F-SQDK--

PEPTIDE SEQ	RAW SIGNAL
QSETFSPDYHKRFHSQ	84 ± 15
SETFSPDYHKRFHSQD	130 ± 3
ETFSPDYHKRFHSQDK	9,987 ± 828
TFSPDYHKRFHSQDKN	9,226 ± 87
FSPDYHKRFHSQDKNM	12,579 ± 349
SPDYHKRFHSQDKNMW	14,205 ± 1,974
PDYHKRFHSQDKNMWV	9,621 ± 4,648
DYHKRFHSQDKNMWVD	9,394 ± 256
YHKRFHSQDKNMWVDM	7,475 ± 14
HKRFHSQDKNMWVDME	8,829 ± 1,829
KRFHSQDKNMWVDMEL	11,451 ± 1,364
RFHSQDKNMWVDMELS	8,612 ± 2,091
FHSQDKNMWVDMELSK	13,451 ± 3,796
HSQDKNMWVDMELSKE	169 ± 57
SQDKNMWVDMELSKEV	303 ± 67

COMPLETE EPITOPE - INCOMPLETE EPITOPE - SIGNAL ABOVE THRESHOLD

Supplementary Figure S14. Detection and mapping of linear epitopes located at different positions in peptides. *The figure shows different examples of epitopes and how the average raw antigenicity signal and its standard deviation vary according to the position of the epitope within assayed 16mer peptides. Examples show that the measured antibody-binding is strong as long as the epitope is present within the peptide, and suggest that in-situ peptide synthesis and yield in the arrays is robust for this peptide length. Complete epitope sequences were derived from the single-residue mutagenesis analysis. Examples were selected from those with a motif length less than 10 amino acids and that were not false positives (see Supplementary Data S8). Data corresponds to samples that were positive for these epitopes (indicated).*

In our analysis of peptide array data we used a very conservative threshold, working under the assumption that positives coming out of this screening would be translated into other immunoassays, e.g. multiwell immunosorbent assays. We have previous experience in translation of candidate peptides from different peptide array platforms to ELISA assays [9–11] and have been mostly successful in translating epitopes using either the best peptide in a peak or other nearby peptides. We usually synthesise and assay only one peptide per antigen, and often these short peptides will be reactive against a few sera but in some cases they may fail to reproduce the expected level of seroprevalence as observed in the arrays using more peptides. Hence, success when translating to FLISA assays is not straightforward. However, by fine mapping antibody-binding peaks at high-resolution (1-residue offset); and having some assessment of quantitative signal plus seroprevalence (population diversity), we have successfully selected and produced antigens either as recombinant proteins or as synthetic peptides. Below we are providing two examples of our experience when moving from arrays to FLISA assays.

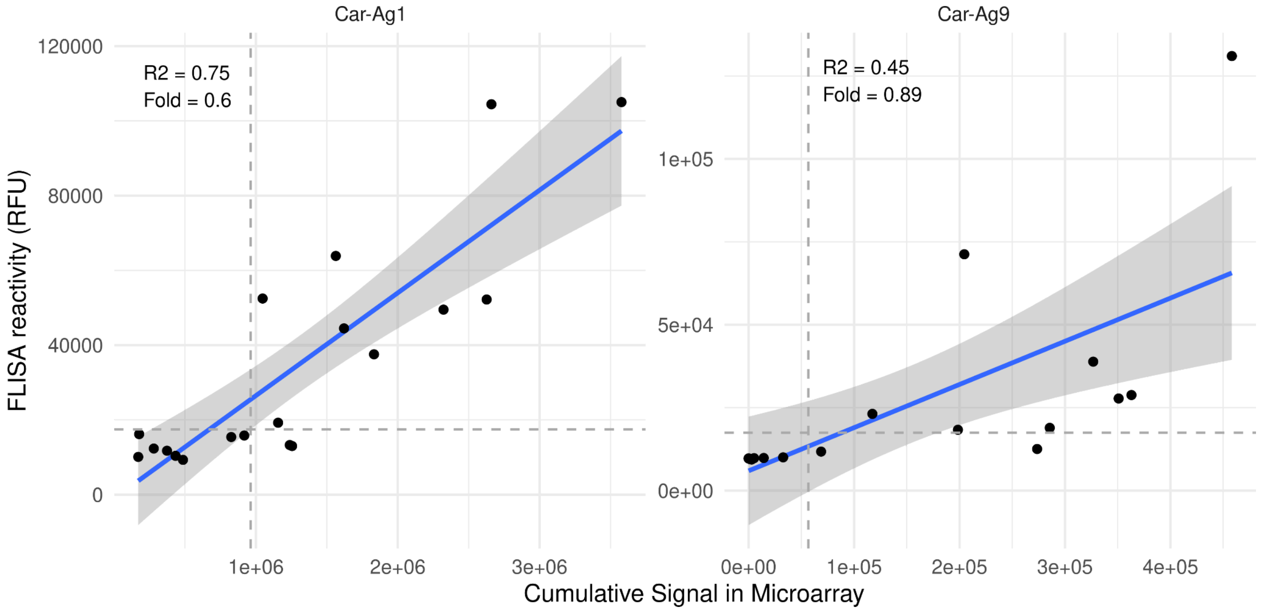

Supplementary Figure S15. Signal correlation between FLISA and microarray experiments. *The figure shows two examples of antigens derived from this work assayed in FLISA format and their correlation with the microarray assay with the same serum samples. CAR-Ag1 (left) was expressed as a recombinant protein containing the corresponding reactive region of the antigen, whereas CAR-Ag9 (right) was synthesised as a short peptide (see Supplementary Data S10 for details). FLISA reactivity (Y-axis) is the average of duplicate assays. Microarray reactivity (X-axis) is the cumulative signal of all individual peptides that span the antigen used in FLISA assays. A linear adjustment is shown in blue with a grey-shadowed zone representing the 95% confidence interval. The R-squared statistic is indicated for each estimation (R2) and the proportion of samples remaining reactive in the FLISA assay is indicated as Fold. Source data are provided as a Source Data file.*

REFERENCES

1. Gruber, A. & Zingales, B. Trypanosoma cruzi: Characterization of Two Recombinant Antigens with Potential Application in the Diagnosis of Chagas′ Disease. *Exp. Parasitol.* **76**, 1–12 (1993).
2. Ibañez, C. F., Affranchino, J. L. & Frasch, A. C. Antigenic determinants of Trypanosoma cruzi defined by cloning of parasite DNA. *Mol Biochem Parasitol* **25**, 175–184 (1987).
3. Ibañez, C. F. *et al.* Multiple Trypanosoma cruzi antigens containing tandemly repeated amino acid sequence motifs. *Mol. Biochem. Parasitol.* **30**, 27–33 (1988).
4. Affranchino, J. L. *et al.* Identification of a Trypanosoma cruzi antigen that is shed during the acute phase of Chagas' disease. *Mol Biochem Parasitol* **34**, 221–228 (1989).
5. Goto, Y., Carter, D. & Reed, S. G. Immunological dominance of Trypanosoma cruzi tandem repeat proteins. *Infect Immun* **76**, 3967–3974 (2008).
6. da Silveira, J. F., Umezawa, E. S. & Luquetti, A. O. Chagas disease: recombinant Trypanosoma cruzi antigens for serological diagnosis. *Trends Parasitol.* **17**, 286–91 (2001).
7. Buschiazzo, A. *et al.* Sequence of the gene for a Trypanosoma cruzi protein antigenic during the chronic phase of human Chagas disease. *Mol. Biochem. Parasitol.* **54**, 125–128 (1992).
8. Newman, A. M. & Cooper, J. B. XSTREAM: A practical algorithm for identification and architecture modeling of tandem repeats in protein sequences. *BMC Bioinformatics* **8**, 382 (2007).
9. Carmona, S. J., Sartor, P. A., Leguizamón, M. S., Campetella, O. E. & Agüero, F. Diagnostic peptide discovery: prioritization of pathogen diagnostic markers using multiple features. *PloS One* **7**, e50748 (2012).
10. Carmona, S. J. *et al.* Towards high-throughput immunomics for infectious diseases: Use of next-generation peptide microarrays for rapid discovery and mapping of antigenic determinants. *Mol. Cell. Proteomics* **14**, (2015).
11. Mucci, J. *et al.* Next-generation ELISA diagnostic assay for Chagas Disease based on the combination of short peptidic epitopes. *PLoS Negl. Trop. Dis.* **11**, e0005972 (2017).
