# Supplementary material for: The Trypanosoma cruzi Antigen and Epitope Atlas: antibody specificities in Chagas disease patients across the Americas
Source: Nat Commun. 2023 Apr 3;14:1850. doi: 10.1038/s41467-023-37522-9 (PMC10070320; doi:10.1038/s41467-023-37522-9)
Supplement: Supplementary file 12 — Description of Additional Supplementary Files [file 41467_2023_37522_MOESM12_ESM.pdf]

Supplementary Data 1
Title: Overview of arrays used in this work
Description: This table shows a diagram of the microarrays used to analyse each design, CHAGASTOPE-v1 and CHAGASTOPE-v2.

Supplementary Data 2
Title: Serum samples and pools
Description: This table shows a list of the human serum samples used in this study.

Supplementary Data 3
Title: CHAGASTOPE array slides and assays
Description: This table shows a detailed list of the assayed arrays.

Supplementary Data 4
Title: *T. cruzi* sequences cross-reactive with normal human serum (healthy subjects, Chagas-negative) **Description:** This table shows all the proteins where the Chagas-negative pooled serum samples reached an antigenicity signal greater than the antigenicity threshold for CHAGASTOPE-v1.

Supplementary Data 5
Title: Best antigenic region per cluster in pooled serum samples
Description: This table shows a representative antigenic region with the highest seroprevalence in CHAGASTOPE-v1 for each selected cluster.

Supplementary Data 6

Title: *T. cruzi* sequences cross-reactive with Leishmaniasis samples

Description: This table shows all the proteins where the Leishmania-positive pooled serum samples reached an antigenicity signal greater than the antigenicity threshold for CHAGASTOPE-v1.

Supplementary Data 7

Title: Best antigenic region per cluster in individual serum samples

Description: This table shows a representative antigenic region with the highest seroprevalence in CHAGASTOPE-v2 for each selected cluster.

Supplementary Data 8

Title: Single-residue mutational scanning results for shown epitopes

Description: This table shows mutagenized peaks corresponding to 232 different sequences analyzed by single mutational scanning and the resulting core residues from each.

Supplementary Data 9

Title: Detailed single-residue mutational scanning results for all epitopes

Description: This table shows mutagenized peaks corresponding to all sequences analyzed by single mutational scanning and the resulting core residues.

Supplementary Data 10

Title: Immunosorbent assays (FLISA) assay details.

Description: Description of CAR-Ag antigens (proteins of origin and sequences) and samples (Origin, sex, and clinical diagnosis) used in fluorescent-linked immunosorbent assays (FLISA).
