# Supplementary material for: The Trypanosoma cruzi Antigen and Epitope Atlas: antibody specificities in Chagas disease patients across the Americas
Source: Nat Commun. 2023 Apr 3;14:1850. doi: 10.1038/s41467-023-37522-9 (PMC10070320; doi:10.1038/s41467-023-37522-9)
Supplement: Supplementary file 14 — Reporting Summary [file 41467_2023_37522_MOESM14_ESM.pdf]

nature portfolio

Corresponding author(s): Fernán Agüero

Last updated by author(s): Mar 11, 2023

Reporting Summary

Nature Portfolio wishes to improve the reproducibility of the work that we publish. This form provides structure for consistency and transparency in reporting. For further information on Nature Portfolio policies, see our Editorial Policies and the Editorial Policy Checklist.

Statistics

For all statistical analyses, confirm that the following items are present in the figure legend, table legend, main text, or Methods section.

n/a	Confirmed	
☐	☒	The exact sample size (*n*) for each experimental group/condition, given as a discrete number and unit of measurement
☐	☒	A statement on whether measurements were taken from distinct samples or whether the same sample was measured repeatedly
☐	☒	The statistical test(s) used AND whether they are one- or two-sided<br>*Only common tests should be described solely by name; describe more complex techniques in the Methods section.*
☒	☐	A description of all covariates tested
☐	☒	A description of any assumptions or corrections, such as tests of normality and adjustment for multiple comparisons
☐	☒	A full description of the statistical parameters including central tendency (e.g. means) or other basic estimates (e.g. regression coefficient) AND variation (e.g. standard deviation) or associated estimates of uncertainty (e.g. confidence intervals)
☒	☐	For null hypothesis testing, the test statistic (e.g. *F*, *t*, *r*) with confidence intervals, effect sizes, degrees of freedom and *P* value noted<br>*Give P values as exact values whenever suitable.*
☒	☐	For Bayesian analysis, information on the choice of priors and Markov chain Monte Carlo settings
☒	☐	For hierarchical and complex designs, identification of the appropriate level for tests and full reporting of outcomes
☐	☒	Estimates of effect sizes (e.g. Cohen's *d*, Pearson's *r*), indicating how they were calculated

Our web collection on statistics for biologists contains articles on many of the points above.

Software and code

Policy information about availability of computer code

Data collection	No custom software or algorithm was used for data collection
Data analysis	Custom software used for data analysis are available at these GitHub Repositories: https://github.com/trypanosomatics/The-Chagas-Disease-Antigen-and-Epitope-Atlas, https://github.com/trypanosomatics/ggseqlogo, DOI: 10.5281/zenodo.7696856. This software was created in R (4.0.3) and requires the following packages (dependencies): data.table (1.13.0), zoo (1.8-9), preprocessCore (1.52.1), dplyr (1.0.2), reshape2 (1.4.4), and pheatmap (1.0.12).

For manuscripts utilizing custom algorithms or software that are central to the research but not yet described in published literature, software must be made available to editors and reviewers. We strongly encourage code deposition in a community repository (e.g. GitHub). See the Nature Portfolio guidelines for submitting code & software for further information.

Data

Policy information about availability of data

All manuscripts must include a data availability statement. This statement should provide the following information, where applicable:

- Accession codes, unique identifiers, or web links for publicly available datasets
- A description of any restrictions on data availability
- For clinical datasets or third party data, please ensure that the statement adheres to our policy

The Peptide Array data generated in this study (raw and processed) have been deposited in the ArrayExpress Functional Genomics Data Collection under accession codes A-MTAB-692 [https://www.ebi.ac.uk/biostudies/arrayexpress/arrays/A-MTAB-692] and A-MTAB-693 [https://www.ebi.ac.uk/biostudies/arrayexpress/arrays/

A-MTAB-693] (Array Designs); and under accession codes E-MTAB-11651 [https://www.ebi.ac.uk/biostudies/arrayexpress/studies/E-MTAB-11651] and E-MTAB-11655 [https://www.ebi.ac.uk/biostudies/arrayexpress/studies/E-MTAB-11655] (Assay Data). The processed peptide data mapped to T. cruzi gene identifiers and antigenicity plots for all proteins are also available at this Figshare Collection: https://doi.org/10.6084/m9.figshare.19991021. Peptide array data can also be explored interactively at the https://chagastope.org website. The data that support the findings of this study are available without restrictions from the sources listed above. Source Data for all data presented in graphs within the manuscript are provided in the Source Data file.

Human research participants

Policy information about studies involving human research participants and Sex and Gender in Research.

Reporting on sex and gender	The manuscript does not report or use sex as a covariate. Information on sex of donors of blood/serum samples is reported.
Population characteristics	The experimental groups were cases (Chagas Disease, infected) and controls (healthy), and the additional covariate analyzed was the geographic origin of the subjects (country, region) used as a proxy to cover different human populations.
Recruitment	Recruitment has been made based on convenience sampling. No compensation was provided to participants.
Ethics oversight	The organizations that approved the study protocol are mentioned in the main text (Methods).

Note that full information on the approval of the study protocol must also be provided in the manuscript.

Field-specific reporting

Please select the one below that is the best fit for your research. If you are not sure, read the appropriate sections before making your selection.

[x] Life sciences [ ] Behavioural & social sciences [ ] Ecological, evolutionary & environmental sciences

For a reference copy of the document with all sections, see nature.com/documents/nr-reporting-summary-flat.pdf

Life sciences study design

All studies must disclose on these points even when the disclosure is negative.

Sample size	Based on previous analyses performed at the lab (Carmona SJ 2015) we determined that a sample size of n=6 per population or group was sufficient for antigen and epitope discovery. Here, we exceeded this sample size (n=11-12 per group, total n=116; 71 positive; 45 negative-control).
Data exclusions	For the discovery screening, we excluded samples with low antibody titres in standard ELISA against T. cruzi. Exclusion criteria were pre-established.
Replication	All microarray assays were performed in duplicate (same biological sample, technical replicates). All replication assays were successful.
Randomization	Samples were allocated into experimental groups based on T. cruzi infection status and geographic origin. Randomization was not relevant.
Blinding	Investigators were not blinded to group allocation during data collection or analysis. Blinding of T. cruzi infection status was not relevant to this study, as there was no experimental treatment or hypothesis being tested. Serum samples were used 'as is' for antigen and epitope discovery and mapping. Blinding during data analysis was not relevant because reactivity of samples to known antigens reveals group allocation (infected vs healthy).

Reporting for specific materials, systems and methods

We require information from authors about some types of materials, experimental systems and methods used in many studies. Here, indicate whether each material, system or method listed is relevant to your study. If you are not sure if a list item applies to your research, read the appropriate section before selecting a response.

Materials & experimental systems

n/a	Involved in the study	
☐	☒	Antibodies
☒	☐	Eukaryotic cell lines
☒	☐	Palaeontology and archaeology
☒	☐	Animals and other organisms
☒	☐	Clinical data
☒	☐	Dual use research of concern

Methods

n/a	Involved in the study	
☒	☐	ChIP-seq
☒	☐	Flow cytometry
☒	☐	MRI-based neuroimaging

Antibodies

Antibodies used

Alexa Fluor® 647-conjugated goat anti-human IgG secondary antibody (Jackson ImmunoResearch #109-605-098). Alexa Fluor® 488-conjugated goat anti-human IgG secondary antibody (Jackson ImmunoResearch #109-545-098).

Validation

Target: Human; Host: Goat; Antibody Format: Whole IgG; Specificity: IgG, Fcγ fragment specific; Minimal Cross Reactivity: Bovine, Horse, Mouse Serum Proteins; Conjugate: Alexa Fluor® 647; Purification: Affinity-Purified Antibodies; Clonality: Polyclonal; RRID: AB_2337889. See https://www.jacksonimmuno.com/catalog/products/109-605-098
Target: Human; Host: Goat; Antibody Format: Whole IgG; Specificity: IgG, Fcγ fragment specific; Minimal Cross Reactivity: Bovine, Horse, Mouse Serum Proteins; Conjugate: Alexa Fluor® 488; Purification: Affinity-Purified Antibodies; Clonality: Polyclonal; RRID: AB_2337840. See https://www.jacksonimmuno.com/catalog/products/109-545-098
